# TLR9 Binding to Beclin 1 and Mitochondrial SIRT3 by a Sodium-Glucose Co-Transporter 2 Inhibitor Protects the Heart from Doxorubicin Toxicity

**DOI:** 10.3390/biology9110369

**Published:** 2020-10-29

**Authors:** Chao-Yung Wang, Chun-Chi Chen, Mei-Hsiu Lin, Hui-Ting Su, Ming-Yun Ho, Jih-Kai Yeh, Ming-Lung Tsai, I-Chang Hsieh, Ming-Shien Wen

**Affiliations:** 1 Department of Cardiology, Chang Gung Memorial Hospital, and Chang Gung University College of Medicine, Taoyuan City 333, Taiwan; mr3228@gmail.com (C.-C.C.); vanisa@ocean.ag (M.-H.L.); keikei@ocean.ag (H.-T.S.); b9005017@hotmail.com (M.-Y.H.); potential.ya@gmail.com (J.-K.Y.); dogofly@gmail.com (M.-L.T.); hsiehic@ms28.hinet.net (I.-C.H.); wenms123@gmail.com (M.-S.W.); 2 Institute of Cellular and System Medicine, National Health Research Institutes, Zhunan 350, Taiwan

**Keywords:** SGLT2 inhibitor, doxorubicin, TLR9, SIRT3, Beclin 1, empagliflozin, heart failure

## Abstract

**Simple Summary:**

We described the existence of an integrated Beclin 1, TLR9, and SIRT3 network involving autophagy, oxidative stress, and mitochondria that is essential for empagliflozin to protect against doxorubicin toxicity in the heart. Empagliflozin treatment increases the abundance of mitochondrial SIRT3 and enhances the activation of TLR9 to bind with Beclin 1, triggering communication to the autophagic, immune system, and inflammatory machinery. From a therapeutic standpoint, SIRT3 loss-of-function variant DCM patients lose the empagliflozin-protective effects, since SIRT3 is implicated in modern world diseases, such as aging, circadian disturbance, and obesity.

**Abstract:**

Large cardiovascular outcome trials have reported favorable effects of sodium-glucose co-transporter 2 (SGLT2) inhibitors on heart failure. To study the potential mechanism of the SGLT2 inhibition in heart failure, we used the murine doxorubicin-induced cardiomyopathy model and identified the toll-like receptor 9 (TLR9), NAD-dependent deacetylase sirtuin-3 (SIRT3), and Beclin 1, acting in a complex together in response to empagliflozin treatment. The interactions and implications in mitochondrial function were evaluated with *TLR9* deficient, *SIRT3* deficient, *Beclin 1* haplodeficient, and autophagy reporter mice and confirmed in a patient with SIRT3 point mutation and reduced enzymatic activity. The SGLT2 inhibitor, empagliflozin, protects the heart from doxorubicin cardiomyopathy in mice, by acting through a novel Beclin 1-toll-like receptor (TLR) 9-sirtuin-(SIRT) 3 axis. TLR9 and SIRT3 were both essential for the protective effects of empagliflozin. The dilated cardiomyopathy patient with SIRT3 point mutation and reduced enzymatic activity is associated with reduced TLR9 activation and the absence of mitochondrial responses in the heart after the SGLT2 inhibitor treatment. Our data indicate a dynamic communication between autophagy and Beclin 1-TLR9-SIRT3 complexes in the mitochondria in response to empagliflozin that may serve as a potential treatment strategy for heart failure.

## 1. Introduction

Heart failure develops in approximately 20% of patients with type 2 diabetes and is associated with increased mortality. Traditional treatment strategies focusing on intensive glucose-lowering effects to reduce heart failure and cardiovascular outcomes have proven to be ineffective [1,2] or even harmful [3]. Accumulating evidence now suggests that the link between diabetes and heart failure lies beyond glucose homeostasis. For example, glucose-lowering by rosiglitazone is associated with an increased risk of heart failure while at the same time achieving the clinical trial targets of glucose control [4]. Diabetic patients with heart failure have associated mitochondrial dysfunction, increased oxidative stress, and increased endoplasmic reticulum stress in their hearts [5]. Several clinical trials of the sodium-glucose co-transporter 2 (SGLT2) inhibitors [6] and a meta-analysis [7] have reported that heart failure patients receiving SGLT2 inhibitors had lower risks of cardiovascular death, regardless of the presence [8] or absence of diabetes [9].

In the Empagliflozin Cardiovascular Outcome Event Trial in Type 2 Diabetes Mellitus Patients—Removal of Excess Glucose (EMPA-REG OUTCOME) trial, the difference between empagliflozin, a highly selective SGLT2 inhibitor (2500-fold compared to SGLT1), and placebo was driven by a significant reduction in hospitalization for heart failure, with no significant between-group difference in the risk of myocardial infarction or stroke [10]. The benefits of SGLT2 inhibition may even extend to heart failure patients without diabetes [9]. These cardiovascular outcome trials of SGLT2 inhibitors have led to numerous speculations and studies related to their potential protective mechanisms in patients with heart failure [11]. Some of the proposed mechanisms are hemodynamic-related, including natriuresis, osmotic diuresis, blood pressure-lowering, and LV remodeling [12]. Other mechanisms are related to more systemic effects [13], including the regulation of myocardial energetics [14], inhibition of sodium-hydrogen exchange [15], adipokines and myokines [16], uric acid homeostasis [17], elevation in erythropoietin levels [18], increases in endothelial progenitor cells [19], protection from doxorubicin [20], and the regulation of autophagy [21]. Despite this, little is currently known about the molecular mechanisms underlying the cardiac protection provided by SGLT2 inhibitors.

Dissecting the exact molecular mechanism of SGLT2 inhibitors is therefore hampered by the diabetic scenario. Moreover, current clinical trials indicate that SGLT2 inhibitors protect against heart failure outcomes even in patients without diabetes. Further studies also showed that empagliflozin can protect doxorubicin-induced heart failure in mice [20]. This evidence clearly indicates that SGLT2 inhibitors have direct cardiac protection mechanisms other than glucose modulation.

Therefore, in order to avoid the complications of impaired glucose metabolism, and to broaden the possible clinical indications, we decided to study the molecular mechanism of SGLT2 inhibitors in doxorubicin-induced heart failure.

## 2. Results

### 2.1. The SGLT2 Inhibitor Empagliflozin Treatment Protects against Doxorubicin-Induced Cardiotoxicity

**Figure 1:**
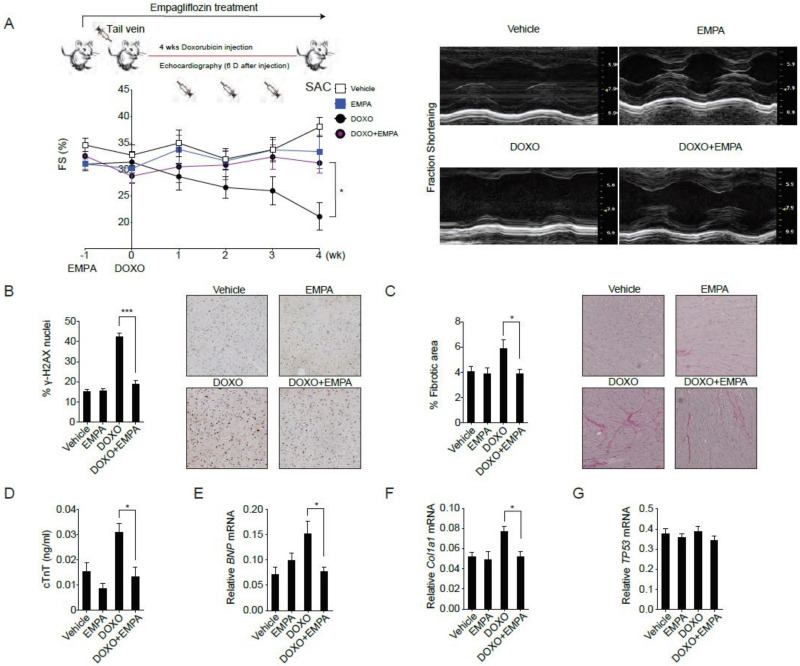
Sodium-glucose co-transporter 2 inhibitor protects against doxorubicin-induced cardiotoxicity. (**A**) C57BL/6 mice were fed with empagliflozin starting from −1 week before weekly intraperitoneal doxorubicin injections for 4 weeks. Left ventricular function determined by m-mode echocardiography at the indicated time points (*n* = 10 for the vehicle or empagliflozin fed mice, 12 for the doxorubicin injection or doxorubicin injection with empagliflozin fed mice. * *p* < 0.05, data were analyzed by two-way analysis of variance (ANOVA) with Tukey post hoc analysis). Right panel: Representative echocardiograms from mice fed with vehicle or empagliflozin and injected with or without doxorubicin. (**B**–**G**) Mice were sacrificed (SAC) immediately following the fourth injection at week 4. (**B**) Phosphorylation of H2A histone family member X (γ-H2AX) staining of left ventricular sections with representative images and quantifications, *** *p* < 0.001. (**C**) Picrosirius red staining with representative images and quantification, * *p* < 0.05. (**D**) Serum cardiac troponin-T, * *p* < 0.05. (**E**) *BNP* mRNA, * *p* < 0.05. (**F**) *Col1a1* mRNA, * *p* < 0.05. (**G**) *TP53* mRNA. (*n* = 10 for the Vehicle or EMPA, 12 for the DOXO or DOXO + EMPA, data were analyzed by one-way ANOVA with Tukey post hoc analysis). Data are represented by mean ± s.e.m. (EMPA, empagliflozin; DOXO, doxorubicin; DOXO + EMPA, doxorubicin and empagliflozin; FS, fraction shortening).

To avoid the confounding influences of body weight loss and decreased food intake and to mimics the clinical usage of doxorubicin, we adapted the preclinical intravenous doxorubicin-induced cardiomyopathy mouse model [22]. Empagliflozin treatment of mice was initiated one week before 4 weeks of doxorubicin injections. Four weeks of doxorubicin injections alone resulted in worse cardiac contractility (Figure 1A), DNA damage (Figure 1B), cardiac fibrosis (Figure 1C), and cardiomyocyte damage (Figure 1D) as measured by serum cardiac troponin T abundances. Compared to mice receiving doxorubicin alone, mice receiving empagliflozin treatment before doxorubicin exhibited better cardiac contractility (Figure 1A), decreased DNA damage (Figure 1B), less cardiac fibrosis (Figure 1C), decreased cardiomyocyte damage (Figure 1D) as well as lower expression levels of BNP (brain natriuretic peptide) (Figure 1E) and Col1a1 (collagen type I, α 1) (Figure 1F) mRNAs. The levels of TP53 mRNA (Figure 1G) were similar in the two treatment groups. These data suggest that empagliflozin exerts beneficial effects on doxorubicin-induced cardiomyopathy. 

### 2.2. SGLT2 Inhibition Increases the Autophagic Flux in Mouse Hearts

**Figure 2:**
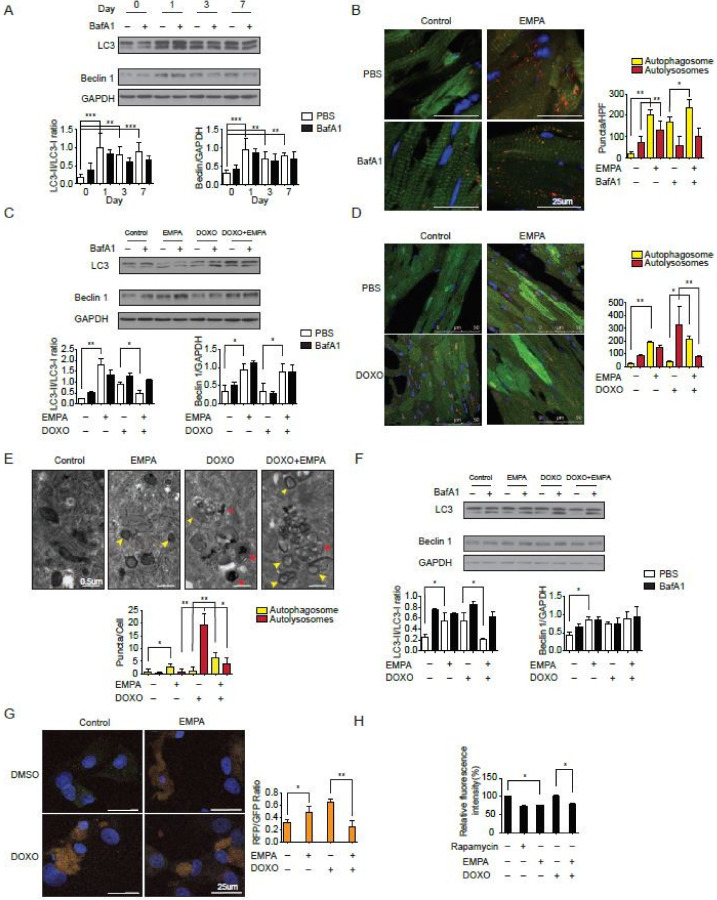
Sodium-glucose co-transporter 2 inhibition increases the autophagic flux in mice hearts. (**A**) Temporal changes in LC3-II/I ratio and Beclin 1 protein abundances after empagliflozin feedings. Hearts were analyzed at different time points after intraperitoneal injection of 1.5 mg/kg BafA1 or normal saline for 2 h (*n* = 3 per group. *** *p* < 0.001, ** *p* < 0.01, data were analyzed by one-way analysis of variance (ANOVA) with Dunnett post hoc analysis). (**B**) Representative fluorescence images of heart sections from CAG–RFP-EGFP–LC3 transgenic mice fed with 7-day empagliflozin and injected with BafA1 or normal saline for 2 h. Quantification of autophagosomes (yellow puncta) and autolysosomes (red puncta) numbers (*n* = 6 mice per group and 10 slices per one mice, ** *p* < 0.01, * *p* < 0.05, data were analyzed by one-way ANOVA with Tukey post hoc analysis). (**C**) Changes in LC3-II/I ratio and Beclin 1 protein abundances after 7-day empagliflozin feedings and 1-dose intravenous doxorubicin injection (5 mg/kg) for 24 h. Hearts were analyzed after intraperitoneal injection of 1.5 mg/kg BafA1 or normal saline for 2 h (*n* = 4 hearts per group with six microscopic fields (14,000 μm^2^) per heart section analyzed. ** *p* < 0.01, * *p* < 0.05, data were analyzed by one-way ANOVA with Tukey post hoc analysis). (**D**) Representative fluorescence images of heart sections from CAG–RFP-EGFP–LC3 transgenic mice injected with doxorubicin (5 mg/kg) and fed with 7-day empagliflozin. Quantification of autophagosomes (yellow puncta) and autolysosomes (red puncta) numbers (*n* = 6 per group. ** *p* < 0.01, * *p* < 0.05, data were analyzed by one-way ANOVA with Tukey post hoc analysis). (**E**) Representative transmission electron microscopy images of cardiomyocytes treated with doxorubicin and empagliflozin. Autophagosomes have two parallel membrane layers separated by a relatively narrower electron-translucent cleft. Autolysosomes have only one membrane and frequently contain electron dense cytoplasmic materials. Quantification of autophagosomes (yellow arrow) and autolysosomes (red arrow) numbers (*n* = 6. * *p* < 0.05, ** *p* < 0.01, data were analyzed by one-way ANOVA with Tukey post hoc analysis). (**F**) Changes in LC3-II/I ratio and Beclin 1 protein abundances in cardiomyocytes treated with doxorubicin and empagliflozin (*n* = 4 per group. * *p* < 0.05, data were analyzed by one-way ANOVA with Tukey post hoc analysis). (**G**) Changes in autophagy flux measured by RFP-GFP-LC3 tandem fluorescent-tagged LC3 in cardiomyocytes treated with doxorubicin and empagliflozin (*n* = 4 per group. * *p* < 0.05, ** *p* < 0.01, data were analyzed by one-way ANOVA with Tukey post hoc analysis). (**H**) Long-lived protein degradation assay in cardiomyocytes (*n* = 4 per group. * *p* < 0.05, data were analyzed by one-way ANOVA with Tukey post hoc analysis). Data are represented by mean ± s.e.m. (BafA1, bafilomycin A1; CAG-RFP-EGFP-LC3, CAG promoter-red fluorescent protein (RFP)–green fluorescent protein-LC3).

Previous evidence has suggested that doxorubicin blocks autophagic flux in cardiomyocytes [22] and that empagliflozin improves autophagy in diabetic animals [21]. To test the hypothesis that the direct molecular mechanism of empagliflozin protection intersects with autophagic signaling in the myocardium, we fed wild-type mice with empagliflozin and analyzed autophagic function in their myocardium. After one day of feeding with empagliflozin, the left ventricles of wild-type mice started to show an increase in the microtubule-associated protein light chain 3-II (LC3-II) to LC3-I ratio (Figure 2A). To determine whether the elevated LC3-II/LC3-I ratio caused by empagliflozin was due to increased autophagosome formation alone or a blockade of autophagic degradation, the LC3-II/LC3-I ratio was analyzed after injection of bafilomycin A1, a lysosomal blockade agent [23]. Further increase in the LC3-II/LC3-I ratio after empagliflozin treatment by bafilomycin A1 would indicate that empagliflozin treatment increases autophagic flux mainly by increasing the formation of autophagosomes. However, after analyzing the LC3-II/LC-I ratio in mice fed with empagliflozin with or without bafilomycin A1, we found that mice injected with bafilomycin A1 showed no further increase in the LC3-II/LC3-I ratio (Figure 2A). This lack of increase in the LC3-II/LC3-I ratio indicates that empagliflozin works through mechanisms other than solely increasing autophagosome formation. There could be several possible reasons as to why the empagliflozin-treated mice showed no changes in the LC3-II/LC3-I ratio after bafilomycin A1 injections. First, empagliflozin could increase the number of autophagosomes by simultaneously increasing both autophagosome formation and blocking autophagic degradation. Second, empagliflozin could interfere with the effects of bafilomycin A1 by restoring the lysosomal function. Third, empagliflozin could have a non-autophagic mechanism that indirectly affects its autophagic activities. To address these points, we used transgenic mice with CAG promoter/enhancer sequences driving LC3 gene expression with dual fluorophores of red fluorescent protein (RFP) and enhanced green fluorescent protein (EGFP) to measure the autophagosome and autolysosome numbers in mice. 

After empagliflozin treatment, the number of autophagosomes in mouse hearts increased 10.2-fold along with a significant increase in the number of autolysosomes (Figure 2B). Bafilomycin A1 injections, which inhibits lysosomal acidification and autophagosome-lysosome fusion, also increased the number of autophagosomes 8.7-fold without increasing the number of autolysosomes. In mice receiving both empagliflozin and bafilomycin A1 injections, the number of autophagosomes was elevated significantly when compared with mice receiving only bafilomycin A1 or mice receiving a placebo. Consistent with the LC3-II/LC3-I ratio data, bafilomycin A1 treatment did not further increase the number of autophagosomes and autolysosomes in the mice treated with empagliflozin. This observation suggests that empagliflozin does indeed increased autophagic flux with both increases in autophagosomes and autolysosomes. However, if the effect of empagliflozin is totally through increasing autophagosome formation, the bafilomycin A1 treatment should further increase the number of autophagosomes and decreases the number of autolysosomes in empagliflozin treated mice. These data suggest that empagliflozin may have other actions beyond enhancing autophagic flux. 

To confirm the increase in autophagic flux seen in mice after doxorubicin treatment, we analyzed the LC3 ratio in mice receiving doxorubicin with or without empagliflozin treatment. After doxorubicin treatment, the LC3-II/LC3-I ratio increased significantly (Figure 2C), similar to that seen in a previous study, which showed that doxorubicin blocks cardiomyocyte autophagic flux by inhibiting lysosome function [22]. In mice that received empagliflozin and doxorubicin, the elevated heart LC3-II/LC3-I ratio caused by doxorubicin was found to be decreased significantly compared to mice that received doxorubicin alone (Figure 2C). Furthermore, in the CAG-RFP-EGFP-LC3 mice, doxorubicin treatment caused an accumulation of autolysosomes, as has previously been reported [22]. In mice treated with both empagliflozin and doxorubicin, there was a significant increase in the number of autophagosomes and a significant decrease in the number of autolysosomes (Figure 2D). These data suggest that empagliflozin not only increases autophagic flux but also relieves the doxorubicin toxicity by decreasing the accumulation of autolysosomes via unknown mechanisms.

We assessed the direct effects of empagliflozin on neonatal rat ventricular cardiomyocytes. Direct treatment of cardiomyocytes with empagliflozin increased the LC3-II/LC3-I ratio in a dose-dependent manner (Figure S1). Consistent with the findings in mice, the transmission electron microscopy showed the accumulation of autolysosomes in doxorubicin-treated cardiomyocytes. After empagliflozin treatment, the cardiomyocytes displayed increases in the number of autophagosomes with decreases in the number of autolysosomes (Figure 2E). Empagliflozin treatment of cardiomyocytes reversed the autophagic dysfunction and decreased the accumulation of autolysosomes caused by doxorubicin as assessed by measurements of the LC3-II/LC3-I ratio (Figure 2F) and by the RFP-EGFP-LC3 reporter (Figure 2G). To further confirm the increased autophagic function, we measured long-lived protein degradation using a fluorescent pulse-chase analysis. Empagliflozin increased long-lived protein turnover in cardiomyocytes similar to rapamycin, a potent autophagy inducer. Long-lived protein turnover was inhibited by doxorubicin and this was reversed by empagliflozin (Figure 2H). Taken together, our findings suggest that empagliflozin does indeed increases autophagic flux in hearts and cardiomyocytes, but its protective effects against doxorubicin can only be partially explained by enhanced autophagy.

### 2.3. Empagliflozin Further Protects against Doxorubicin Cardiotoxicity in Beclin 1 Deficient Mice

**Figure 3:**
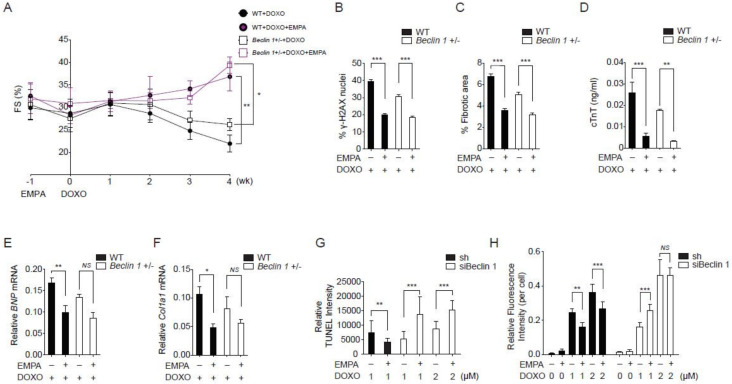
Empagliflozin further protects doxorubicin cardiotoxicity in Beclin 1 deficient mice. (**A**) Left ventricular function determined by m-mode echocardiography at the indicated time points (*n* = 7 per group. * *p* < 0.05, ** *p* < 0.01, data were analyzed by two-way analysis of variance (ANOVA) with Tukey post hoc analysis). (**B–F**) Mice were sacrificed immediately following the fourth injection at week 4. (**B**) Phosphorylation of H2A histone family member X (γ-H2AX) staining of left ventricular sections, *** *p* < 0.001. (**C**) Picrosirius red staining, *** *p* < 0.001. (**D**) Serum cardiac troponin-T, *** *p* < 0.001, ** *p* < 0.01. (**E**) *BNP* mRNA, ** *p* < 0.01, *p* = 0.06 for Beclin 1+/− + DOXO vs. Beclin 1+/− + DOXO/EMPA. (**F**) *Col1a1* mRNA, * *p* < 0.05, *NS*, not significant (*n* = 7 per group, data were analyzed by one-way ANOVA with Tukey post hoc analysis). (**G**) TUNEL staining of the neonatal cardiomyocytes. (*n* = 4, ** *p* < 0.01, *** *p* < 0.001, data were analyzed by the Kruskal-Wallis one-way ANOVA). (**H**) Cellular ROS detected by H_2_DCFDA fluorescence (*n* = 4, ** *p* < 0.01, *** *p* < 0.001, *NS*, not significant, data were analyzed by the two-way ANOVA with Tukey post hoc analysis). Data are represented by mean ± s.e.m. (WT, wild-type; EMPA, empagliflozin; DOXO, doxorubicin; ROS, reactive oxygen species).

Previous studies have shown that Beclin 1 haplodeficient mice are protected from doxorubicin cardiotoxicity by an attenuation of the initiation of autophagy and a decreased demand for lysosomal processing [22]. We reasoned that if empagliflozin protects against doxorubicin cardiotoxicity through both autophagic and non-autophagic mechanisms, it may provide further protection in Beclin 1 haplodeficient mice. Beclin 1 haplodeficient mice had better left ventricular function without statistically significance when compared to wild-type mice after doxorubicin injections. Empagliflozin treatment of Beclin 1 haplodeficient mice further improved left ventricular functions when compared to Beclin 1 haplodeficient mice not treated with empagliflozin after doxorubicin injections (Figure 3A). In addition, treatment of empagliflozin in Beclin 1 haplodeficient mice further ameliorated DNA damage (Figure 3B), cardiac fibrosis (Figure 3C), and cardiomyocyte injury (Figure 3D). Treatment of Beclin 1 haplodeficient mice with empagliflozin also decreased BNP and Col1a1 mRNA expression levels (Figure 3E,F). The fact that empagliflozin provided protective effects against doxorubicin cardiomyopathy in Beclin 1 haplodeficient mice has two possible explanations. First, empagliflozin elicits its effects through Beclin 1-independent mechanisms. Second, empagliflozin works through Beclin 1 to provide its protective effects. Our data showed that empagliflozin had a trend towards increasing Beclin 1 abundances in the heart (Figure 2A,C,F), which theoretically would attenuate the protective effects of Beclin 1 haplodeficiency. Therefore, empagliflozin may work with Beclin 1 through a more complex mechanism. 

To delineate between these two possibilities and because Beclin 1 homozygous knockout mice are non-viable, we knocked down Beclin 1 levels to less than 10% in cardiomyocytes (Figure S2). In cardiomyocytes with a near absence of Beclin 1, treatment with empagliflozin and doxorubicin paradoxically increased the apoptosis rate (Figure 3G) and the levels of reactive oxygen species (ROS, Figure 3H). These data significantly decrease the possibility that the empagliflozin protection mechanisms is Beclin 1-independent. We decided to further explore the Beclin 1 non-autophagic mechanism of empagliflozin.

### 2.4. Empagliflozin Increases the Binding of TLR9 to Beclin 1 and to SIRT3

**Figure 4:**
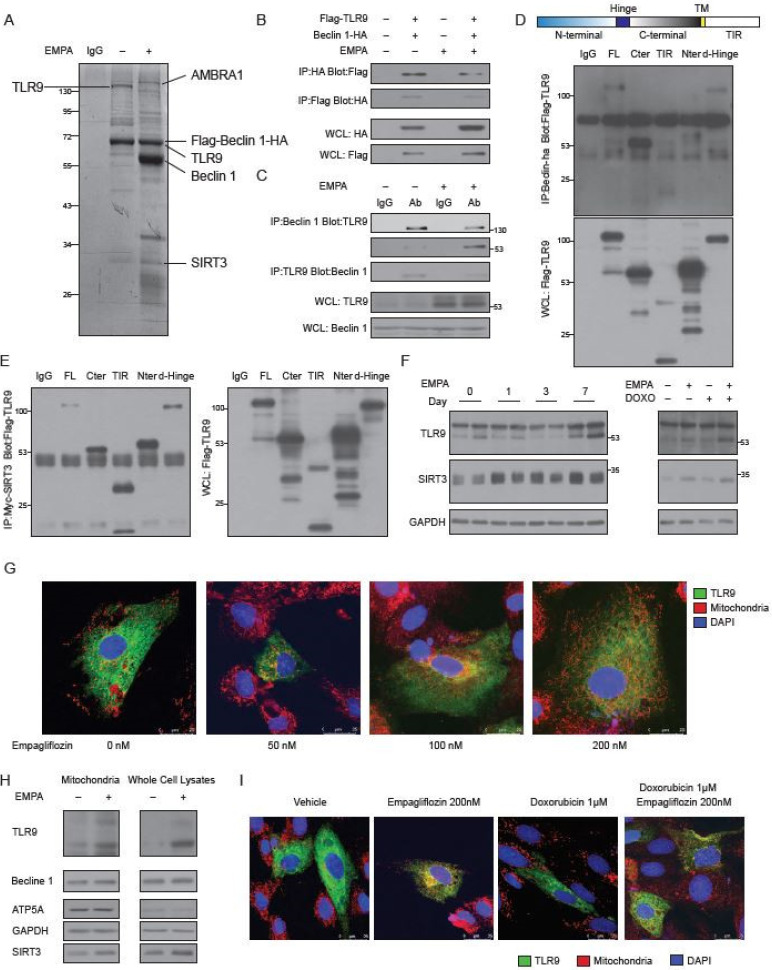
Identification of TLR9 and SIRT3 as major empagliflozin-regulated components of the Beclin 1 complexes. (**A**) SYPRO Ruby staining of affinity-purified Beclin 1 complexes from Flag-Beclin 1-HA overexpressed 293 cell lines treated with vehicles (lane 2) or empagliflozin (lane 3). Specific Beclin 1-interacting protein bands were analyzed by mass spectrometry. (**B**) Coimmunoprecipitation of Beclin 1 with TLR9 or TLR9 with Beclin 1 from 293 cells with or without empagliflozin (200 nM) treatment. Western blot analysis of 293 whole-cell extracts transfected with Flag-TLR9 and Beclin 1-HA and immunoprecipitated with Beclin 1-HA (lane 1) or Flag-TLR9 (lane 2). (**C**) Beclin 1 interaction with TLR9 in mice hearts and regulated by empagliflozin. WT mice were fed with or without empagliflozin for 7 days. Heart lysates were immunoprecipitated with Beclin 1 or TLR9 antibody recognizing full-length 130 kDa or cleaved form 53 kDa and analyzed by western blot. (**D**) Protein domains involved in the Beclin-TLR9 interaction. Flag-tagged TLR9 and its indicated fragments (FL: full length TLR9; Cter: C-terminal TLR9; TIR: TIR domain of the TLR9; Nter: N-terminal TLR9; d-Hinge: full length TLR9 without Hinge domain) were coexpressed in 293 cells with Beclin 1-HA, and anti-HA immunoprecipitates were analyzed by anti-Flag immunoblotting. (**E**) Protein domains involved in the TLR9-SIRT3 interaction. Flag-tagged TLR9 and its indicated fragments (FL: full length TLR9; Cter: C-terminal TLR9; TIR: TIR domain of the TLR9; Nter: N-terminal TLR9; d-Hinge: full length TLR9 without Hinge domain) were coexpressed in 293 cells with Myc-Sirt3, and anti-Myc immunoprecipitates were analyzed by anti-Flag immunoblotting. (**F**) Left: Temporal changes in TLR9 and SIRT3 protein abundances after empagliflozin feedings. Hearts were analyzed at different time points after empagliflozin feedings. Right: Western blot analysis of changes in TLR9 and SIRT3 protein abundances after intravenous doxorubicin injections (5 mg/kg) and 7-day empagliflozin feedings. (**G**) Images of AC16 human cardiomyocytes stained for mitochondria (red), nucleus (blue), and overexpressed GFP-TLR9 (green). Cells were treated with indicated doses of empagliflozin before imaging. Increases of the orange signals (green + red) indicate the increases of mitochondrial localization of TLR9. (**H**) Western blot analysis of the mitochondria and whole-cell lysates from AC16 human cardiomyocytes treated with vehicle or empagliflozin (200 nM). (**I**) Images of AC16 human cardiomyocytes stained for mitochondria (red), nucleus (blue), and overexpressed GFP-TLR9 (green). Cells were treated with indicated doses of empagliflozin before imaging.

To identify the empagliflozin-regulated molecular factors that are related to Beclin 1, we used a dual epitope-tagging strategy to isolate Beclin 1 protein complexes from cells treated with or without empagliflozin. Cell extracts of human embryonic kidney cells expressing the Beclin 1 protein containing an N-terminal Flag and C-terminal HA epitopes were sequentially subjected to affinity chromatography on Flag agarose beads and HA-affinity columns (Figure 4A). As expected, we identified Flag-Beclin 1-HA as a major component of the complexes. In the absence of empagliflozin treatment, two protein bands of ~130 kDa and ~28 kDa from the Beclin 1 complexes contained peptide sequences corresponding to the toll-like receptor 9 (TLR9) and NAD-dependent deacetylase sirtuin-3 (SIRT3) proteins, respectively. After empagliflozin treatment, we identified three prominent bands of ~140 kDa (corresponding to Autophagy and Beclin 1 Regulator 1 (AMBRA1)), ~60 kDa (corresponding to native Beclin 1), and ~28 kDa (corresponding to SIRT3). The ~64 kDa Flag-Beclin 1-HA band also contained peptide sequences derived from the TLR9 protein. The increased abundances of AMBRA1 and native Beclin 1 in the Beclin 1 complexes after empagliflozin treatment are, therefore, consistent with our previous data that empagliflozin enhances autophagic flux (Figure 2A) [24]. SIRT3 is located in the mitochondria and has been implicated in regulating cellular respiration, ROS, and thermogenesis [25]. TLR9 is an important receptor that is expressed in immune cells. After binding to viral or bacterial DNA, TLR9 triggers pro-inflammatory reactions and activates innate immunity. However, the molecular connection between Beclin 1 and TLR9 mediated inflammatory responses is poorly understood.

To confirm the physical interaction between Beclin 1 and TLR9, we overexpressed full-length Flag-TLR9 and HA-Beclin 1 in H9c2 cells. Flag-full-length-TLR9 was clearly detected in the immunoprecipitations obtained with the anti-HA-Beclin 1 antibody but not with a control IgG. Conversely, HA-Beclin 1 was readily immunoprecipitated by the anti-Flag antibody, but not with a control antibody (Figure 4B, Figure S3A). We noticed that the binding of the full-length TLR9 to the Beclin 1 was decreased after empagliflozin treatment. To clarify the binding differences between full-length and cleaved TLR9 [26] to Beclin 1 in mice, we immunoprecipitated Beclin 1 from wild-type mouse hearts and found that native TLR9 could be detected in the complexes (Figure 4C). Using an antibody specific for full-length TLR9 and an antibody for the cleaved and active form of TLR9, we showed that empagliflozin increased the bindings of cleaved TLR9 and decreased the bindings of full-length TLR9 to Beclin 1 in mouse hearts (Figure 4C). 

To identify which domain within TLR9 interacts with Beclin 1, we have made different Flag-tagged TLR9 domain variants, such as full length TLR9 (FL), TLR9 with only c-terminal domain (Cter), TLR9 with only TIR domain (TIR), TLR9 with only N-terminal domain (Nter), and full length TLR9 without Hinge domain (d-Hinge). We expressed different Flag-tagged TLR9 protein variants along with HA-tagged Beclin 1 and analyzed their interaction by immunoprecipitations. The Cter TLR9, which mimics the cleaved TLR9, had the strongest binding to Beclin 1 (Figure 4D). TLR9 proteins Nter or TIR did not bind to Beclin 1. These data indicate that TLR9 binds to Beclin 1 through its C-terminus and that empagliflozin enhances the binding of the active and cleaved form of TLR9 to Beclin 1 in mouse hearts.

After confirming the binding of TLR9 and Beclin 1, we next addressed the role of SIRT3 in this complex. Overexpression of SIRT3, but not SIRT3H248Y (a catalytic mutant of SIRT3 lacking deacetylase activity) increased the binding of Beclin 1 and TLR9 (Figure S3A). SIRT3 co-immunoprecipitated with TRL9 in cells (Figure 4E) and bound to it directly (Supplementary Figure S3B). Unlike the binding to Beclin 1, which limited to Cter TLR9, the interaction between SIRT3 and TLR9 involved FL, Cter, TIR, Nter, and d-Hinge variants. Immunoprecipitation of the Myc-SIRT3 was able to pull down the Beclin 1-HA (Figure S3C). However, the in vitro binding assay failed to detect the direct binding between SIRT3 and Beclin 1 (results not shown). These findings suggest that empagliflozin increases the bindings of SIRT3 to the Beclin 1-TLR9 complex through TLR9. Since SIRT3 mostly exists in mitochondria [25], the increased SIRT3 abundances in the Beclin 1-TLR9 complex after empagliflozin treatment suggests that the Beclin 1-TLR9 complex mainly functions in the mitochondria. 

### 2.5. Empagliflozin Increases the TLR9 Activation in Mitochondria

After one-week empagliflozin treatment, the abundances of TLR9 and SIRT3 were significantly increased in the heart (Figure 4F, left panel). These increases in the abundances of cleaved TLR9 and SIRT3 were also observed in mice injected with doxorubicin after empagliflozin treatment (Figure 4F, right panel). 

To test the possibility that empagliflozin increases the TLR9 in the mitochondria, we treated cardiomyocytes with empagliflozin. In the absence of empagliflozin treatment, TLR9 was localized in the cytoplasm with minimal colocalization with the mitochondria. After treatment with empagliflozin, significantly more TLR9 was colocalized with the mitochondria, indicating the increases of TLR9 in the mitochondria (Figure 4G, Figure S4A); this was confirmed by the quantitative western blotting (Figure 4H). We also noticed that empagliflozin treatment increased the abundance of SIRT3 in the mitochondria but not of Beclin 1 (Figure 4H). The increases of TLR9 in the mitochondria induced by empagliflozin was also observed in the doxorubicin treated cardiomyocytes (Figure 4I, Figure S4B). 

### 2.6. SIRT3 is Indispensable for the Mitochondrial TLR9 Trafficking and Function by Empagliflozin

**Figure 5:**
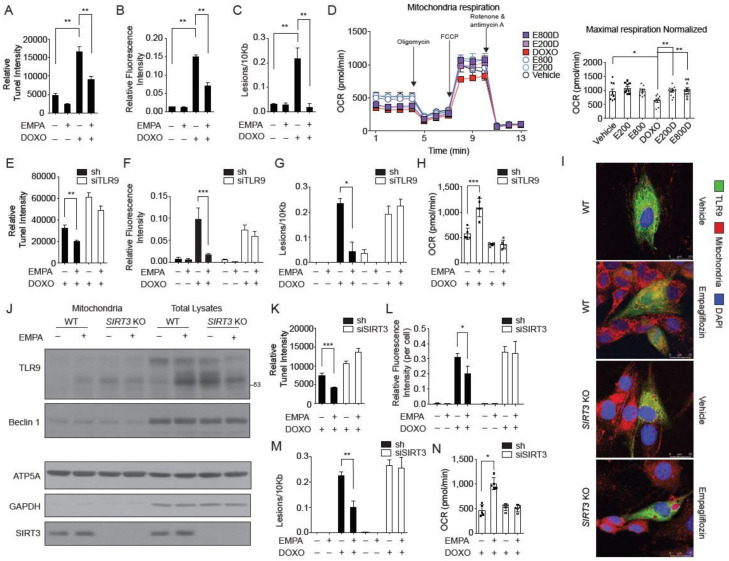
SIRT3 is indispensable for the mitochondrial TLR9 trafficking and function by empagliflozin. (**A**) TUNEL staining of the neonatal cardiomyocytes treated with empagliflozin (200 nM) and doxorubicin (1 μM) for 24 h (*n* = 6, ** *p* < 0.01, data were analyzed by the Kruskal-Wallis one-way ANOVA). (**B**) Cellular ROS detected by H_2_DCFDA fluorescence (*n* = 4, ** *p* < 0.01, data were analyzed by the one-way ANOVA with Tukey post hoc analysis). (**C**) Mitochondrial DNA damages (*n* = 4, ** *p* < 0.01, data were analyzed by the one-way ANOVA with Tukey post hoc analysis). (**D**) Mitochondrial respiration analyzed by Seahorse instruments. Maximal respiration normalized by cell numbers (*n* = 4, * *p* < 0.05, ** *p* < 0.01, data were analyzed by the one-way ANOVA with Tukey post hoc analysis). E800D and E200D = empagliflozin 800 nM or 200 nM with doxorubicin 1 μM; E800 and E200 = empagliflozin 800 nm or 200 nM. (**E**) TUNEL staining of the neonatal cardiomyocytes transfected with TLR9 siRNA or sh control (*n* = 4, ** *p* < 0.01, data were analyzed by the Kruskal-Wallis one-way ANOVA). (**F**) Cellular ROS detected by H_2_DCFDA fluorescence (*n* = 4, *** *p* < 0.001, data were analyzed by the two-way ANOVA with Tukey post hoc analysis). (**G**) Mitochondrial DNA damages. (*n* = 4, * *p* < 0.05, data were analyzed by the two-way ANOVA with Tukey post hoc analysis). (**H**) Maximal respiration normalized by cell numbers (*n* = 4, ** *p* < 0.01, data were analyzed by the one-way ANOVA with Tukey post hoc analysis). (**I**) Images of WT and *SIRT3* KO cells stained for mitochondria (red), nucleus (blue), and overexpressed GFP-TLR9 (green). Cells were treated with empagliflozin (200 nM) before imaging. (**J**) Western blot analysis of the mitochondria and total lysates from hearts of WT and *SIRT3* KO mice fed with or without empagliflozin. (**K**) TUNEL staining of the neonatal cardiomyocytes transfected with SIRT3 siRNA or sh control (*n* = 4, *** *p* < 0.001, data were analyzed by the Kruskal-Wallis one-way ANOVA). (**L**) Cellular ROS detected by H_2_DCFDA fluorescence (*n* = 4, * *p* < 0.05, data were analyzed by the two-way ANOVA with Tukey post hoc analysis). (**M**) mitochondrial DNA damages assay with empagliflozin (200 nM) and doxorubicin (1 μM). (*n* = 6, ** *p* < 0.01, data were analyzed by the two-way ANOVA with Tukey post hoc analysis). (**N**) Maximal respiration analyzed by Seahorse instruments (*n* = 4, * *p* < 0.05, data were analyzed by the one-way ANOVA with Tukey post hoc analysis). Data are represented by mean ± s.e.m. (EMPA, empagliflozin; DOXO, doxorubicin; OCR, oxygen consumption rate; ROS, reactive oxygen species; WT, wild-type; *SIRT3* KO, *SIRT3* knockout).

The observation that empagliflozin increased TLR9 activation in the mitochondria raised three possibilities. First, empagliflozin could exert its protection of doxorubicin toxicity by modulating mitochondrial function. We showed that empagliflozin treatment reversed the doxorubicin-induced apoptosis in cardiomyocytes (Figure 5A). Treatment with empagliflozin also reduced the excessive ROS levels (Figure 5B) and DNA damages (Figure 5C) caused by doxorubicin. Consistent with these findings, empagliflozin treatment was able to rescue the depressed mitochondrial respiration caused by doxorubicin in cardiomyocytes (Figure 5D). However, treatment with empagliflozin did not affect glycolysis, fatty acid oxidation, palmitate uptake, or carnitine palmitoyltransferase-1 activity (Figure S5).

Second, TLR9, which has previously been known to traffic from the endoplasmic reticulum to the Golgi and endosomal network, also localizes to the mitochondria and protects cardiomyocytes from doxorubicin toxicity. In cardiomyocytes, knockdown of TLR9 abolished the empagliflozin protective effects on apoptosis (Figure 5E), ROS levels (Figure 5F), and DNA damage (Figure 5G) caused by doxorubicin treatment. The effects of mitochondrial respiration restoration by empagliflozin were also significantly diminished in TLR9-knockdown cardiomyocytes (Figure 5H). Thus, our data showed that TLR9 is not only increased in the mitochondria but is also responsible for the empagliflozin-mediated protection from doxorubicin in cardiomyocytes. 

Third, previous studies have shown that SIRT3 is only expressed in the nucleus and mitochondria. Accordingly, we questioned if empagliflozin-induced mitochondrial TLR9 increases is SIRT3-dependent. The increased TLR9 in the mitochondria by empagliflozin treatment was significantly dampened in both SIRT3 knockdown (Figure S6) and SIRT3 knockout cells (Figure 5I, Figure S4C). To confirm this observation, we determined whether the mitochondrial increases of TLR9 induced by empagliflozin treatment in mouse hearts is also SIRT3-dependent. After one week of empagliflozin treatment, the mitochondria in mouse hearts showed a significant increase in the abundances of TLR9 (Figure 5J). In SIRT3 knockout mice, there was a significantly higher accumulation of TLR9 in the heart without empagliflozin treatment. Unlike in wild-type mice, empagliflozin treatment did not increase the TLR9 in the mitochondria in SIRT3 knockout mice (Figure 5J), indicating that SIRT3 is indispensable for the mitochondrial regulation of TLR9 in mice. These data indicated that SIRT3 may play a critical role in the mechanism by which empagliflozin protects against doxorubicin cardiotoxicity. Knockdown of SIRT3 in cardiomyocytes abolished the protective effect of empagliflozin on apoptosis (Figure 5K), excess ROS levels (Figure 5L), DNA damage (Figure 5M), and reduced mitochondrial respiration (Figure 5N) caused by doxorubicin treatment. In summary, SIRT3 is critical in recruiting TLR9 to the mitochondria and is essential in the mechanism by which empagliflozin protects against doxorubicin cardiotoxicity.

### 2.7. SIRT3 and TLR9 are Essential for the Empagliflozin Protection for Doxorubicin Toxicity

**Figure 6:**
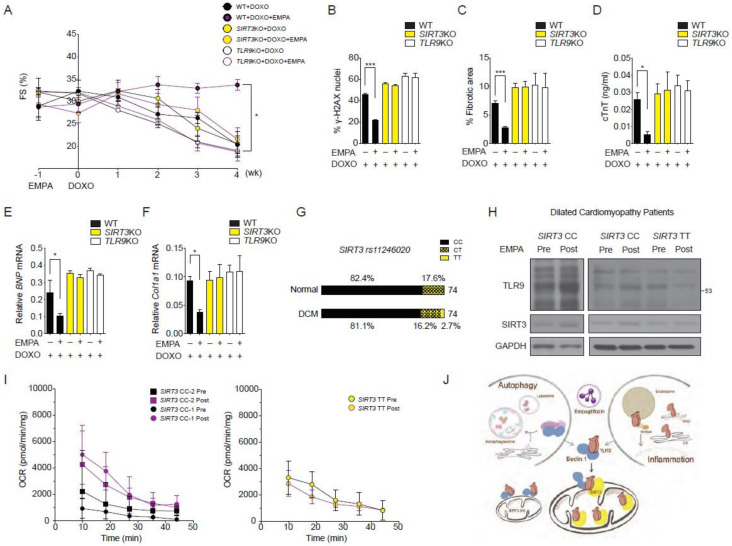
Reduction of empagliflozin effects in *SIRT3* or *TLR9* knockout mice and in humans with *SIRT3* point mutation with reduced enzymatic activity. (**A**) Left ventricular function determined by m-mode echocardiography at the indicated time points (*n* = 7 per group. * *p* < 0.05 for WT + DOXO + EMPA vs. *SIRT3*KO + DOXO + EMPA or *TLR9*KO + DOXO + EMPA, data were analyzed by two-way analysis of variance (ANOVA) with Tukey post hoc analysis). (**B**–**F**) Mice were sacrificed at 4 weeks after doxorubicin injection. (**B**) Phosphorylation of H2A histone family member X (γ-H2AX) staining of left ventricular sections, *** *p* < 0.001. (**C**) Picrosirius red staining, *** *p* < 0.001. (**D**) Serum cardiac troponin-T, * *p* < 0.05. (**E**) *BNP* mRNA, * *p* < 0.05. (**F**) *Col1a1* mRNA, * *p* < 0.05 (*n* = 7 per group, data were analyzed by one-way ANOVA with Tukey post hoc analysis). (**G**) Graphic representation of the SNP *rs11246020* frequency in the prospective cohort of patients with DCM or control patients. CC corresponds to the normal alleles, CT to SNP heterozygosity, and TT to SNP homozygosity. (**H**) Western blots for TLR9 and SIRT3 performed on the cardiac biopsies before and after 28 days of empagliflozin treatment from patients with DCM (2 with *SIRT3 rs11246020* CC and 1 with TT genotypes). (**I**) Basal mitochondrial respiration performed on the cardiac biopsies before and after 28 days of empagliflozin treatment from patients with DCM (2 with *SIRT3 rs11246020* CC and 1 with TT genotypes). (**J**) Schematic of the empagliflozin promotes binding of the Beclin 1-TLR9 and trafficking to mitochondria toward SIRT3. In *SIRT3* knockout mice, empagliflozin cannot enhance the trafficking of TLR9 toward mitochondria. Data are represented by mean ± s.e.m. (WT, wild-type, EMPA, empagliflozin; DOXO, doxorubicin, DCM, dilated cardiomyopathy).

To determine whether the in vivo cardioprotective effects of empagliflozin depend on SIRT3 and TLR9, we injected doxorubicin into SIRT3 and TLR9 knockout mice and treated them with empagliflozin. Notably, the empagliflozin protective effect on the reduced left ventricular function caused by doxorubicin that was observed in wild-type mice was not observed in SIRT3 or TLR9 knockout mice (Figure 6A). The lack of a beneficial empagliflozin effect in SIRT3 and TLR9 knockout mice was also seen for DNA damage levels (Figure 6B), cardiac fibrosis levels (Figure 6C), cardiac troponin-T levels (Figure 6D), BNP mRNA levels (Figure 6E), and Col1a1 mRNA levels (Figure 6F). Collectively, these data indicated that empagliflozin protects against doxorubicin-induced cardiomyopathy through a mitochondrial TLR9-SIRT3 mechanism.

### 2.8. Effects of Empagliflozin in Human with SIRT3 Point Mutation and Reduced Enzymatic Activity

Finally, we investigated the SIRT3-dependent mitochondrial increases of TLR9 by empagliflozin in subjects with dilated cardiomyopathy (DCM). We focused on the non-synonymous point mutation encoded by rs11246020 (NC_000011.10:g.233067C > T), which results in a mutation of amino acid 208 from valine to isoleucine in SIRT3 and results in a 34% decrease in the SIRT3 catalytic efficiency [27]. We screened 74 DNA samples from our prospective cohort of patients with dilated cardiomyopathy and 74 DNA samples from age- and gender-matched control healthy subjects (Table S1). In normal subjects, the distributions of the CC, CT, and TT genotypes were 61, 31, and 0. In the DCM cohort, the distributions were 60, 12, and 2 (Figure 6G). rs11246020 was not associated with the occurrence of DCM occurrence in either recessive, co-dominant, or dominant models. We excluded DCM patients with diabetes mellitus and recruited two DCM patients with the wild-type CC genotype and one DCM patient with a homozygous TT genotype to examine the effects of empagliflozin.

These three DCM patients received empagliflozin at a dose of 25 mg once daily for 28 days. Patients were evaluated before empagliflozin treatment and on treatment day 28 with an assessment of heart failure, volume status, renal function, echocardiography, and endomyocardial biopsy. Using the biopsy samples, we determined the TLR9 protein abundance in the heart by western blotting. In the two DCM patients with the CC genotype, empagliflozin increased cardiac TLR9 protein abundance, while in the DCM patient with the TT genotype, empagliflozin treatment did not increase the TLR9 abundance (Figure 6H). Analysis of the baseline mitochondrial respiration of the endomyocardial biopsy samples showed that the two DCM patients with the CC genotype had an increased mitochondrial respiration rate in response to empagliflozin treatment. The DCM patient with the TT genotype had no response to empagliflozin on the baseline mitochondrial respiration rate (Figure 6I). These results suggest that activation of TLR9 and an enhanced mitochondrial respiration rate in the heart induced by empagliflozin are hampered by the SIRT3 loss-of-function polymorphism in humans.

## 3. Discussion

Here, we showed the existence of an integrated Beclin 1, TLR9, and SIRT3 network involving autophagy, oxidative stress, and mitochondria that is essential for the ability of empagliflozin to protect against doxorubicin toxicity in the heart. As shown in the model in Figure 6J, empagliflozin treatment increases the abundance of mitochondrial SIRT3 (Figure 4F or Figure 5J) and enhances the activation of TLR9 to bind with Beclin 1, triggering communication to the autophagic, immune system, and inflammatory machinery. The increased abundances of SIRT3 then direct the Beclin 1-TLR9 complex to traffic toward the mitochondria, where the activated TLR9 enhances the mitochondrial respiration rate and exerts its protection against ROS and apoptosis. Of particular interest from a therapeutic standpoint is the finding that the SIRT3 knockout mice and the SIRT3 loss-of-function variant DCM patient both lose the empagliflozin protective effects, since SIRT3 is implicated in many modern world diseases, such as aging, circadian disturbance, pulmonary hypertension, and obesity [25,28]. Recent findings also identified that TLR9 and Beclin 1 crosstalk regulates muscle function and glucose metabolism during exercise [29].

The action of empagliflozin cannot be totally attributed to an autophagic mechanism. The reasons for this are as follows: first, we noticed that in CAG-RFP-EGFP-LC3 mice, empagliflozin treatment followed by doxorubicin injections simultaneously increased the number of autophagosomes and decreased the number of autolysosomes. Doxorubicin blocks autophagic flux by inhibiting lysosome acidification resulting in the accumulation of lysosomes. For empagliflozin to decrease the number of autolysosomes, it must act through other pathways rather than only by increasing autophagic flux and enhancing autophagosome formation. Second, the empagliflozin treatment also protects against doxorubicin toxicity in Beclin 1 haploinsufficient mice. Third, empagliflozin increases the binding of active TLR9 and SIRT3 to the Beclin 1, indicating that empagliflozin has a Beclin 1-related autophagic mechanism and a TLR9-SIRT3-related non-autophagic mechanism. Although previous studies and our data support the fact that empagliflozin can enhance autophagic flux, our evidence suggests that empagliflozin acts through Beclin 1 with both autophagic and autophagic-independent mechanisms.

In our experiments, empagliflozin treatment increased the binding of activated TLR9 to Beclin 1 and enhanced the TLR9 associated with SIRT3 in the mitochondria. We have noticed that after empagliflozin treatment, the abundances of mitochondrial TLR9 increased significantly but not the abundances of mitochondrial Beclin 1 (Figure 5J). This finding suggests that the mitochondrial effects of empagliflozin are mainly mediated through TLR9 and confirms a previous controversial role of TLR9 in mitochondria [30,31]. These earlier reports suggest that TLR9 can reduce energy substrates, increase stress tolerance [32], and modulate mitochondrial ATP synthesis [31] in cardiomyocytes. TLR9 can also prevent cardiac rupture after myocardial infarction [33]. Knockout of TLR9 in the sarcoplasmic/endoplasmic reticulum Ca2+ ATPase 2a (SERCA2a) diastolic heart failure mice reduces their survival [34]. However, ablation of TLR9 in mice attenuates myocardial ischemia injury [35]. This discrepancy may be explained by the tissue-specific functions of the TLR9. The TLR9 ligand-receptor system in innate immunity can concomitantly send different signals to immune cells and nonimmune cells. Upon tissue injury, immune cells induce inflammatory cascades, whereas cardiomyocytes increase stress tolerance and mitochondrial function. Although SGLT2 inhibitors provide significant cardiac and renal protection, there is a possible adverse event for lower limb amputation, which requires an FDA boxed warning [36]. The exact mechanism and significance of the amputation associated with SGLT2 inhibitors are still controversial. A previous study has shown that SGLT2 inhibition can inhibit endothelial cell proliferation and tube formation [37]. TLR9 activation can also suppress angiogenesis, impair endothelial regeneration, and promote atherosclerosis [38,39]. The different tissue actions of SGLT2 inhibition could, therefore, be partially explained by the versatile roles of TLR9.

The role of SIRT3 in models of cardiac disease is also under intensive investigation. We found that Beclin 1 and TLR9 are both important for the empagliflozin-mediated mitochondrial protection against doxorubicin. Knockout of SIRT3 in mice or in a patient with the SIRT3 loss-of-function polymorphism also greatly diminished the empagliflozin effects on mitochondria. These results suggest that the canonical Beclin 1 autophagy machinery cooperates with the adaptive innate immunity TLR9 signaling to deliver the cellular needs to the mitochondria and regulate mitochondrial function with SIRT3. Given the remarkable tissue-specificity of these signaling events and their different physiological responses, further analysis of the functionality of this Beclin 1-TLR9-SIRT3 machinery in other tissues or diseases is clearly needed.

Beclin 1 is at the center of the empagliflozin therapeutic effects and the doxorubicin cardiac toxicity. Beclin 1 complexes adapt dynamically with different binding co-factors to perform various physiological functions, such as autophagy, apoptosis, and inflammation [29]. The autophagy-independent functions of Beclin 1 have been under intense scrutiny recently and have been found to be important in many human diseases. The binding of Beclin 1 to the TLR9, which belongs to the pattern-recognition receptors of the innate immune system, opens several possibilities and future study directions. We hypothesize that doxorubicin modifies the Beclin 1 complexes moiety and function to exert its cardiac toxicity in many aspects, which can be reversed by the empagliflozin modification of the Beclin 1 complexes.

These findings led to the question if the Beclin 1-TLR9-SIRT3 axis could explain the other clinical effects of SGLT2 inhibitors in the EMPA-REG, CANVAS, or DAPA-HF trials. SGLT2 inhibitors may increase erythropoiesis via enhanced erythropoietin secretion [18], decrease serum uric acid [40], and reduces myocardial ketone utilization [41]. One possibility is that these effects reflect the different tissue-specific functions of the Beclin 1-TLR9 complex. A second possibility is that the empagliflozin protective effects work through different signaling events in various conditions. This appears to be the case for the effects of metformin [42,43]. Moreover, as current SGLT2 inhibitors also provide various degrees of SGLT1 inhibition, the effects of SGLT1 inhibition cannot be ignored. Therefore, it is important to explore differences between empagliflozin, a highly selective SGLT2 inhibitor with a ~2500-fold selectivity over SGLT1, and sotagliflozin, a dual SGLT inhibitor with a ~20-fold selectivity over SGLT1 in their protective effects against doxorubicin. 

Our study has limitations. First, we performed the study in nondiabetic animals and humans, hampering our ability to explore and extend these findings to the diabetic animals and patients. We advocate studying the Beclin 1-TLR9-SIRT3 axis in diabetic populations to address future clinical usages and therapeutic options. Second, we did not assess any of TRL9 stimulated inflammatory markers such as NF-kB and TNF-α. This could largely limit the interpretation or implication of the study. Third, SIRT3 has deacetylation activity that mediates the changes in the acetylation of proteins. It is possible that SIRT3 regulates the proteins involved in the Beclin 1-TLR9-SIRT3 axis that are proposed to be responsible for empagliflozin mediated processes following increased SIRT3 levels. Fourth, the empagliflozin treatment begins before doxorubicin injections, we cannot rule out the possibility that empagliflozin has a direct or indirect interaction with doxorubicin, which would reduce the toxic effects of doxorubicin. Fifth, we have only recruited two DCM patients with the wild-type CC genotype and one DCM patient with a homozygous TT genotype. More patients will be needed for confirmatory conclusions.

Finally, the discovery of the collaboration between Beclin 1 and TLR9, their ability to modulate mitochondrial function and ROS, and the critical role of SIRT3 in the mitochondria provide the most compelling proof of principle that SGLT2 inhibition protects the heart directly.

## 4. Materials and Methods

### 4.1. Cells, Reagents, and Antibodies

AC16 human cardiomyocyte cells (SCC109) were from Merck and cultured in DMEM/F12 with 2 mM L-Glutamine and 12.5% fetal bovine serum. Neonatal rat ventricular myocytes were isolated from less than 3-day-old Sprague-Dawley rats with Pierce primary cardiomyocyte isolation kit (88281, ThermoFisher Scientific, Waltham, MA, USA). H9c2 (CRL-1446, American Type Culture Collection, Manassas, VA, USA) cells were cultured in DMEM with 10% fetal bovine serum.

Empagliflozin for mice treatment was from Boehringer Ingelheim. Empagliflozin (sc-482194) for cell experiments was from ChemCruz Biochemicals, Dallas, TX, USA. Doxorubicin (d1515) was from Sigma-Aldrich, St. Louis, MO, USA. Bafilomycin A1 (88899-44-2) was from Cayman Chemical, Ann Arbor, MI, USA. Rapamycin (ab120224) was from Abcam, Cambridge, UK. The anti-LC3 antibody (ab48394, Abcam), anti-Beclin 1 (#3495, Cell Signaling, Danvers, MA, USA; NB500-249, Novus, Littleton, NH, USA), anti-GAPDH (#2118, Cell Signaling), anti-SIRT3 (#5490S, Cell Signaling), anti-TLR9 (ab17236, ab211012, Abcam; SC-47723, Santa Cruz; NBP2-24729, Novus), anti-ATP5A (ab110273, Abcam), anti-γ-H2AX (ab11174, Abcam), anti-Flag (M2-A8592, Sigma-Aldrich), and anti-HA(12013819001, Roche, Basel, Switzerland) were used. The anti-TLR9, anti-SIRT3, and anti-Beclin 1 antibodies were verified using siRNA knockdown or knockout mice lysates before uses (Figure S2).

### 4.2. Plasmids

The pcDNA3-TLR9-YFP (Doug Golenbock, 13642), pcDNA4-Beclin 1-HA (Qing Zhong, 24399), pcDNA4-Myc-HisB-SIRT3 (Toren Finkel, 24918), and pcDNA4-Myc-HisA-H248Y-Sirt3 (Toren Finkel, 24917) were obtained from Addgene, Watertown, NY, USA. We used CloneAmp HiFi PCR Premix (639298, Clontech, Kusatsu, Japan) to amplify Flag-Beclin 1, full-length TLR9, and corresponding TLR9 segments. The PCR products were then cloned into pCMVTag4B or pcDNA4-HA by In-Fusion HD Cloning Kit (638910, Clontech). The cloned plasmid was transformed into ECOS-101(DH5a) competent cell (LYE678-10, YB biotech). The retroviruses for monitoring autophagy flux were made from pMRX-IP-GFP-LC3-RFP (Noboru Mizushima, 84573, Addgene, Watertown, NY, USA) and pMRX-IP-GFP-LC3-RFP (Noboru Mizushima, 84572, Addgene).

### 4.3. Mice

Mice were maintained on a 12-h light/12-h dark cycle (lights on at 7:00 a.m. and off at 7:00 p.m.). *CAG-RFP-EGFP-LC3* transgenic mice (027139), *Becn1^+/−^*(018429) mice, and *SIRT3*-knock-out (027975) mice were from Jackson Laboratory. *TLR9*-knock-out mice were a generous gift of Dr. Shih-Jen Liu (National Health Research Institutes, Taiwan). Wild-type and homozygous knockout mice were generated by breeding heterozygous male and female mice. All animal experiments were approved by the Chang Gung Memorial Hospital and the Chang Gung University Institutional Animal Care and Use Committee (#109-066, 107-145, 108-142, 014-192) and strictly adhered to the guidelines for animal experiments. 

### 4.4. Animal Experiments

For empagliflozin feeding, male mice 8 to 12 weeks old were fed with standard rodent chow diet, containing 500 mg empagliflozin per 1 kg diet. During our experiments, the average food intake of mice was 2.7 g/day and the estimated empagliflozin ingested was 0.05 mg/g/day for 5 weeks. After one-week of empagliflozin feeding, mice were randomized on the basis of body weight and food intake into the control or doxorubicin group. The doxorubicin (5 mg/kg) was injected via the tail vein once per week for 4 weeks. The control group was injected with normal saline. After each doxorubicin injection for 6 days, mice were anesthetized with isoflurane and received echocardiographic examination (Vevo 2100, VisualSonics, Minato, Japan). Left ventricular fractional shortening was calculated by the left ventricular end-diastolic dimension minus end-systolic dimension divided by the end-diastolic dimension. Serum troponin T (CSB-EL024016MO, Cusabio, Houston, TX, USA) was measured after 4 weeks of doxorubicin injection. The bafilomycin A1 was injected 2 h before the study intraperitoneally (1.5 mg/kg). Mice were euthanized in the fed state from 8:30 a.m. to 12:00 a.m. to minimize the circadian variations.

### 4.5. Histology, Immunohistochemistry, and Immunofluorescence

For tissue samples, mice were euthanized and immediately perfused with 4% PFA in PBS for picrosirius red staining (24901, Polysciences, Taipei, Taiwan) and γ-H2AX staining. The images were obtained with a panoramic histology camera after staining from at least seven sections per mice. For fluorescence experiments, cells were placed on a chamber slide (PEZGS0416, Millipore, Burlington, VT, USA). Before observation, cells were washed with phosphate-buffered saline (PBS) and fixed with 4% paraformaldehyde (PFA) in PBS for 10 min. For fluorescence quantification for mice tissues, frozen sections with OCT were used. For staining mitochondria in cells, Mitotracker Deep Red FM (M22426, Invitrogen, Carlsbad, CA, USA) was used.

### 4.6. RNA Analysis

Cells or Tissues were stored in RNAlater (AM7021, ThermoFisher Scientific, Waltham, MA, USA). RNA was extracted with TRI Reagent (AM9738, ThermoFisher Scientific, Waltham, MA, USA) and was reverse-transcribed. qPCR was performed with a 7900HT system (Applied Biosystems, ThermoFisher Scientific, Waltham, MA, USA) or iQ5 system (Bio-Rad, Hercules, CA, USA) and normalized to 18S rRNA or 36B4. The siRNA was generated by Dharmacon, Lafayette, LA, USA. The assay was independently repeated at least three times.

### 4.7. Protein Analysis

Protein was isolated from homogenized cells or tissues with a cell lysis buffer (#9803, Cell Signaling). The lysates were separated by electrophoresis, transferred to polyvinylidene fluoride membranes, and probed with specific antibodies. The results were normalized to the glyceraldehyde-3-phosphate dehydrogenase band and calculated with ImageJ (NIH, Version 1.52e).

### 4.8. Autophagy Reporter Analysis

For measuring cardiomyocytes autophagy flux, the fluorescence probe, GFP-LC3-RFP-LC3ΔG, was retrovirally introduced into cells. When this probe was expressed in cells, RFP-LC3ΔG cannot undergo lipidation and was stably served as an internal control. The GFP-LC3 was degraded after fusion with lysosomes. The RFP/GFP signal ratio was calculated and correlated with autophagic activity [44]. To analyze the empagliflozin effects on autophagy, the cells were examined in the fed state. For the *CAG-RFP-EGFP-LC3* transgenic mice, RFP fluorescence is stable in autolysosomes, and the combined fluorescence of GFP and RFP yields a yellow signal in autophagosomes in mouse hearts. 

### 4.9. Transmission Electron Microscopy

Hearts were perfused and fixed with 1.5% glutaraldehyde and 1.5% paraformaldehyde in 0.1 M cacodylate buffer. After three washes with 0.1 M cacodylate buffer, hearts were postfixed in 1% osmium tetroxide and 1.5% potassium ferrocyanide (1 h, room temperature). After three rinses with water, specimens were dehydrated with increasing concentrations of ethanol, infiltrated with Epon (14120, EMS, Hatfield, PA, USA), and polymerized in a 60 °C oven overnight. Cells were fixed in 2.5% glutaraldehyde and postfixed in 1% osmium tetraoxide. They were then dehydrated using alcohol and embedded in epoxy resin for sectioning. Ultra-thin sections were stained with uranyl acetate and lead citrate. The tissues and cells were examined with a Hitachi H-7500 transmission electron microscope. 

### 4.10. Long-Lived Protein Degradation Assay

The protein degradation was performed with nonradioactive l-azidohomoalanine (AHA) labeling [45]. The cells were cultured in six-well plates and then rinsed with 2 mL of PBS twice. The natural methionine was depleted with 2 mL of l-methionine-free DMEM with 10% dialyzed FBS for 30 min. The 50 μM AHA was added with 2 mL of l-methionine-free culture DMEM with 10% dialyzed FBS for 18 h. Then AHA was chased out with DMEM with 10% FBS containing l-methionine (2 mM) for 2 h. Then, the cells were added with empagliflozin, doxorubicin, or rapamycin (ab120224, Abcam) for the indicated time and fixed with 4% formaldehyde. The cells were processed with click reaction (50 μM TAMRA azide, 1 mM TCEP, 100 μM TBTA, and 1 mM CuSO4 in PBS) and analyzed by flow cytometry. 

### 4.11. Purification and Identification of Beclin 1 Complexes after Empagliflozin Treatment

To obtain Flag-Beclin 1-HA-expressing cells, 293 cells were transfected with pcDNA4-Flag-Beclin 1-HA. Cells (5 × 10^9^) were treated with PBS or empagliflozin (200 nM) for 18 h and lysed in lysis buffer (25 mM Tris pH 7.4, 150 mM NaCl, 1% NP-40, 1 mM EDTA, 5% glycerol). The extract was diluted with 20 mM HEPES (pH 7.9) and 1 mM EDTA and ultracentrifuged at 25,000 rpm for 2 h at 4 °C. The supernatant was immunoprecipitated using anti-Flag M2 agarose. The precipitate was eluted using the Flag peptide and immunopurified using anti-HA antibody-conjugated agarose [46]. After eluting the anti-HA agarose with HA peptides, we resolved the elutes on a 4–20% gradient gel for SYPRO Ruby staining analysis. Protein bands were subjected to concerted matrix-assisted laser desorption ionization (MALDI) peptide mass fingerprinting and collision-induced fragmentation MS/MS analysis by the Core Facilities for Proteomics (ThermoFisher/Q Exactive HF) located at the Chang Gung Memorial Hospital. 

### 4.12. Mitochondrial Isolation, TUNEL Assay, ROS Detection, and DNA Damages Analysis

Mitochondria were isolated using Mitochondria isolation kit for cells (89874, ThermoFisher Scientific) or tissues (89801, ThermoFisher Scientific). The purity of mitochondria was verified with ATP5A protein analysis. A TUNEL assay was performed according to the DeadEnd Colorimetric TUNEL System (G7130, Promega, Madison, IL, USA). The reactive oxygen species were detected with H_2_DCFDA (D399, Invitrogen). For mitochondrial DNA damage, the DNA was extracted Genomic-tip 20/G (10223, QIAGEN, Hilden, Germany) and Genomic DNA buffer set kit (19060, QIAGEN). The PCR primer used for amplification were rat 14.3-kb mitochondrial genomes (Forward 5′-ATATTTATCACTGCTGAGTCCCGTGG-3′, Reverse 5′-AATTTCGGTTGGGGTGACCTCGGAG-3′) and rat 211-bp mitochondria fragment (Forward 5′-CCTCCCATTCATTATCGCCGCCCTTGC-3′, Reverse 5′-GTCTGGGTCTCCTAGTAGGTCTGGGAA-3′). The quantitative PCR was done with LA PCR Kit, Version 2.1 (RR013A, Clontech, Kusatsu, Japnan) and Quant-iT PicoGreen dsDNA Kit (P7589, Invitrogen, Carlsbad, CA, USA).

### 4.13. Mitochondrial Respiration

The XF96 Extracellular Flux Analyzer (Seahorse Bioscience, North Billerica, MA, USA) was used to measure the oxygen consumption rate in mitochondria freshly isolated from mice or in cells. Oligomycin (1 μM), FCCP (Carbonyl cyanide-4-(trifluoromethoxy)phenylhydrazone, 0.75 μM), and rotenone/antimycin A (1 μM) were used to determine the maximal respiration, proton leak, ATP production, and spare respiratory capacity. 

### 4.14. Clinical Trial Design and Procedures

We prospectively screened patients admitted with heart failure symptoms at the Chang Gung Memorial Hospital from September 2013 to October 2019. After initial clinician evaluations, patients who were more than 20 years old and had a clinical diagnosis of dilated cardiomyopathy were enrolled in this study. Dilated cardiomyopathy was diagnosed according to the European Society of Cardiology criteria. Health participants without a history of heart diseases and with normal LVEF confirmed by echocardiography served as the normal control subjects. The clinical endpoints were specified as all-cause mortality, cardiovascular mortality, non-fatal stroke, heart failure re-hospitalization, or ventricular arrhythmia requiring implantable cardioverter-defibrillator implantation. All participants were followed up in outpatient clinics at 3, 6, 9, 12, 24, and 36 months after the study enrollment. The major cardiac events that occurred during the first visit to enrollment were also included. 

In the second study, dilated cardiomyopathy patients were evaluated by the physician to see whether add-on of the empagliflozin was suitable clinically. Patients with uncontrolled heart failure, renal function impairment, and liver function impairment were excluded. After evaluation, the patients were enrolled in the empagliflozin treatment study. Patients in the treatment study received the pre-treatment cardiac biopsy from internal jugular veins with Bioptome. Right ventricular biopsies were sent for histology, protein, RNA, and mitochondrial analysis immediately. Patients then received the empagliflozin treatment for one month and received a second cardiac biopsy. After each cardiac biopsy, echocardiography was done to ensure no biopsy-related complications and evaluate cardiac functions. The study protocol was approved by the Chang Gung Memorial Hospital Institutional Review Board, and all participants provided written informed consent. 

### 4.15. SNP Genotyping Assay

DNA was extracted from 5 mL of blood using the DNeasy blood kit (69506, QIAGEN, Hilden, Germany). Sequencing of *SIRT3 rs11246020* was performed with a TaqMan assay by using an ABI Prism Sequence Detector 7000 (Applied Biosystems) according to the manufacturer’s protocols. Hardy–Weinberg equilibrium within each group was tested and was found to be nonsignificant for all gene polymorphisms (*p* > 0.05). The genotyping results were reconfirmed by performing polymerase chain reaction (PCR) analysis and direct sequencing.

### 4.16. Statistical Analysis

All statistical analyses were conducted with GraphPad Prism Software v.8.3. Data are presented as mean ± s.e.m. The sample size for each experiment is reported in the text and figure legends. No statistical method was used to predetermine the sample size of the animal studies. The sample sizes were chosen on the basis of our previous experience with wild-type animals receiving doxorubicin injections. There was no blinding for the experiments. Statistical outliers (not more than one per experiment) were determined with the two-sided Grubbs’ outlier test and excluded. We evaluated statistical significance by analysis of variances; differences between groups were tested by one-way ANOVA with post hoc Tukey test or by Kruskal-Wallis test with post hoc Dunn’s test for non-normal distributions. To compare more than two groups of mice with analysis at different time points, a two-way ANOVA with post hoc Tukey test was used.

### 4.17. Data Availability

All requests for raw and analyzed data and materials are promptly reviewed by the Chang Gung University (Material Transfer office) to verify if the request is subject to any intellectual property or confidentiality obligations. Any data and materials that can be shared will be released via a Material Transfer Agreement. 

## 5. Conclusions

This is the first report to investigate the potential mechanism which protects doxorubicin-induced cardiotoxicity by empagliflozin through Beclin 1-TLR9-Sirt3 axis in mitochondria. This mechanism reveals a molecular pathway that may provide future therapeutic targets for cardiac protection in patients receiving oncologic treatments. 

## Supplementary Materials

The following are available online at https://www.mdpi.com/2079-7737/9/11/369/s1, Figure S1: Sodium-glucose co-transporter 2 inhibition increases the autophagic flux in cardiomyocytes, Figure S2: Verification of antibody and efficiency of siBeclin 1, siSirt3, and siTLR9, Figure S3: SIRT3 increased Beclin 1 and TLR9 bindings, Figure S4: Colocalization analysis of overexpressed GFP-TLR9 and mitochondria stained with Mitotracker Deep Red FM, Figure S5: Glycolysis, Fatty acid uptake, and CPT 1 activity in cardiomyocytes after empagliflozin treatment, Figure S6: SIRT3 knockdown and TLR9 abundances after empagliflozin treatment in cardiomyocytes, Table S1: siRNA and Real time PCR primer sequences.

## References

[1] Green J.B., Bethel M.A., Armstrong P.W., Buse J.B., Engel S.S., Garg J., Josse R.G., Kaufman K.D., Koglin J., Korn S. (2015). Effect of Sitagliptin on Cardiovascular Outcomes in Type 2 Diabetes. N. Engl. J. Med..

[2] Castagno D., Baird-Gunning J., Jhund P.S., Zoccai G.B., Macdonald M.R., Petrie M.C., Gaita F., McMurray J.J. (2011). Intensive glycemic control has no impact on the risk of heart failure in type 2 diabetic patients: Evidence from a 37,229 patient meta-analysis. Am. Heart J..

[3] Boussageon R., Bejan-Angoulvant T., Saadatian-Elahi M., Lafont S., Bergeonneau C., Kassaï B., Erpeldinger S., Wright J.M., Gueyffier F., Cornu C. (2011). Effect of intensive glucose lowering treatment on all cause mortality, cardiovascular death, and microvascular events in type 2 diabetes: Meta-analysis of randomised controlled trials. BMJ.

[4] Komajda M., McMurray J.J., Beck-Nielsen H., Gomis R., Hanefeld M., Pocock S.J., Curtis P.S., Jones N.P., Home P.D. (2010). Heart failure events with rosiglitazone in type 2 diabetes: Data from the RECORD clinical trial. Eur. Heart J..

[5] Jia G., Hill M.A., Sowers J.R. (2018). Diabetic Cardiomyopathy: An Update of Mechanisms Contributing to This Clinical Entity. Circ. Res..

[6] Mahaffey K.W., Jardine M.J., Bompoint S., Cannon C.P., Neal B., Heerspink H.J., Charytan D.M., Edwards R., Agarwal R., Bakris G. (2019). Canagliflozin and Cardiovascular and Renal Outcomes in Type 2 Diabetes Mellitus and Chronic Kidney Disease in Primary and Secondary Cardiovascular Prevention Groups. Circulation.

[7] Udell J.A., Yuan Z., Rush T., Sicignano N.M., Galitz M., Rosenthal N. (2018). Cardiovascular Outcomes and Risks After Initiation of a Sodium Glucose Cotransporter 2 Inhibitor: Results From the EASEL Population-Based Cohort Study (Evidence for Cardiovascular Outcomes With Sodium Glucose Cotransporter 2 Inhibitors in the Real World). Circulation.

[8] Zinman B., Wanner C., Lachin J.M., Fitchett D.H., Bluhmki E., Hantel S., Mattheus M., Devins T., Johansen O.E., Woerle H.J. (2015). Empagliflozin, Cardiovascular Outcomes, and Mortality in Type 2 Diabetes. N. Engl. J. Med..

[9] McMurray J.J.V., Solomon S.D., Inzucchi S.E., Køber L., Kosiborod M.N., Martinez F.A., Ponikowski P., Sabatine M.S., Anand I.S., Bělohlávek J. (2019). Dapagliflozin in Patients with Heart Failure and Reduced Ejection Fraction. N. Engl. J. Med..

[10] Fitchett D., Zinman B., Wanner C., Lachin J.M., Hantel S., Salsali A., Johansen O.E., Woerle H.J., Broedl U.C., Inzucchi S.E. (2016). Heart failure outcomes with empagliflozin in patients with type 2 diabetes at high cardiovascular risk: Results of the EMPA-REG OUTCOME® trial. Eur. Heart J..

[11] Verma S. (2019). Potential Mechanisms of Sodium-Glucose Co-Transporter 2 Inhibitor-Related Cardiovascular Benefits. Am. J. Cardiol..

[12] Hallow K.M., Helmlinger G., Greasley P.J., McMurray J.J.V., Boulton D.W. (2018). Why do SGLT2 inhibitors reduce heart failure hospitalization? A differential volume regulation hypothesis. Diabetes Obes. Metab..

[13] Vaduganathan M., Butler J. (2019). SGLT-2 inhibitors in heart failure: A new therapeutic avenue. Nat. Med..

[14] Verma S., Rawat S., Ho K.L., Wagg C.S., Zhang L., Teoh H., Dyck J.E., Uddin G.M., Oudit G.Y., Mayoux E. (2018). Empagliflozin Increases Cardiac Energy Production in Diabetes: Novel Translational Insights Into the Heart Failure Benefits of SGLT2 Inhibitors. JACC Basic Transl. Sci..

[15] Bell R.M., Yellon D.M. (2018). SGLT2 inhibitors: Hypotheses on the mechanism of cardiovascular protection. Lancet Diabetes Endocrinol..

[16] Xu L., Ota T. (2018). Emerging roles of SGLT2 inhibitors in obesity and insulin resistance: Focus on fat browning and macrophage polarization. Adipocyte.

[17] Zhao Y., Xu L., Tian D., Xia P., Zheng H., Wang L., Chen L. (2017). Effects of sodium-glucose co-transporter 2 (SGLT2) inhibitors on serum uric acid level: A meta-analysis of randomized controlled trials. Diabetes Obes. Metab..

[18] Mazer C.D., Hare G.M., Connelly P.W., Gilbert R.E., Shehata N., Quan A., Teoh H., Leiter L.A., Zinman B., Jüni P. (2020). Effect of Empagliflozin on Erythropoietin Levels, Iron Stores and Red Blood Cell Morphology in Patients with Type 2 Diabetes and Coronary Artery Disease. Circulation.

[19] Hess D.A., Terenzi D.C., Trac J.Z., Quan A., Mason T., Al-Omran M., Bhatt D.L., Dhingra N., Rotstein O.D., Leiter A.L. (2019). A novel effect of SGLT2 inhibition to increase circulating pro-vascular progenitor cells in patients with type 2 diabetes. Cell Metab..

[20] Oh C.M., Cho S., Jang J.-Y., Kim H., Chun S., Choi M., Park S., Ko Y.-G. (2019). Cardioprotective Potential of an SGLT2 Inhibitor Against Doxorubicin-Induced Heart Failure. Korean Circ. J..

[21] Aragón-Herrera A., Feijóo-Bandín S., Santiago M.O., Barral L., Campos-Toimil M., Gil-Longo J., Pereira T.M.C., García-Caballero T., Rodríguez-Segade S., Rodríguez J. (2019). Empagliflozin reduces the levels of CD36 and cardiotoxic lipids while improving autophagy in the hearts of Zucker diabetic fatty rats. Biochem. Pharmacol..

[22] Li D.L., Wang Z.V., Ding G., Tan W., Luo X., Criollo A., Xie M., Jiang N., May H., Kyrychenko V. (2016). Doxorubicin Blocks Cardiomyocyte Autophagic Flux by Inhibiting Lysosome Acidification. Circulation.

[23] Mauvezin C., Neufeld T.P. (2015). Bafilomycin A1 disrupts autophagic flux by inhibiting both V-ATPase-dependent acidification and Ca-P60A/SERCA-dependent autophagosome-lysosome fusion. Autophagy.

[24] Nixon R.A. (2013). The role of autophagy in neurodegenerative disease. Nat. Med..

[25] Hirschey M.D., Shimazu T., Goetzman E., Jing E., Schwer B., Lombard D.B., Grueter C.A., Harris C., Biddinger S.B., Ilkayeva O.R. (2010). SIRT3 regulates mitochondrial fatty-acid oxidation by reversible enzyme deacetylation. Nature.

[26] Ewald S.E., Lee B.L., Lau L., Wickliffe K.E., Shi G.-P., Chapman H.A., Barton G.M. (2008). The ectodomain of Toll-like receptor 9 is cleaved to generate a functional receptor. Nature.

[27] Hirschey M.D., Shimazu T., Jing E., Grueter C.A., Collins A.M., Aouizerat B., Stančáková A., Goetzman E., Lam M.M., Schwer B. (2011). SIRT3 Deficiency and Mitochondrial Protein Hyperacetylation Accelerate the Development of the Metabolic Syndrome. Mol. Cell.

[28] Peek C.B., Affinati A.H., Ramsey K.M., Kuo H.-Y., Yu W., Sena L.A., Ilkayeva O., Marcheva B., Kobayashi Y., Omura C. (2013). Circadian Clock NAD+ Cycle Drives Mitochondrial Oxidative Metabolism in Mice. Science.

[29] Liu Y., Nguyen P.T., Wang X., Zhao Y., Meacham C.E., Zou Z., Bordieanu B., Johanns M., Vertommen D., Wijshake T. (2020). TLR9 and beclin 1 crosstalk regulates muscle AMPK activation in exercise. Nature.

[30] Oka T., Hikoso S., Yamaguchi O., Taneike M., Takeda T., Tamai T., Oyabu J., Murakawa T., Nakayama H., Nishida K. (2012). Mitochondrial DNA that escapes from autophagy causes inflammation and heart failure. Nature.

[31] Shintani Y., Drexler H.C.A., Kioka H., Terracciano C.M.N., Coppen S.R., Imamura H., Akao M., Nakai J., Wheeler A.P., Higo S. (2014). Toll-like receptor 9 protects non-immune cells from stress by modulating mitochondrial ATP synthesis through the inhibition of SERCA2. EMBO Rep..

[32] Shintani Y., Kapoor A., Kaneko M., Smolenski R.T., D’Acquisto F., Coppen S.R., Harada-Shoji N., Lee H.J., Thiemermann C., Takashima S. (2013). TLR9 mediates cellular protection by modulating energy metabolism in cardiomyocytes and neurons. Proc. Natl. Acad. Sci. USA.

[33] Omiya S., Omori Y., Taneike M., Protti A., Yamaguchi O., Akira S., Shah A.M., Nishida K., Otsu K. (2016). Toll-like receptor 9 prevents cardiac rupture after myocardial infarction in mice independently of inflammation. Am. J. Physiol. Heart Physiol..

[34] Dhondup Y., Sjaastad I., Sandanger Ø., Aronsen J.M., Ahmed M.S., Attramadal H., Finsen A.V., Zhang L., Ranheim T., Alfsnes K. (2017). Toll-Like Receptor 9 Promotes Survival in SERCA2a KO Heart Failure Mice. Mediat. Inflamm..

[35] Kitazume-Taneike R., Taneike M., Omiya S., Misaka T., Nishida K., Yamaguchi O., Akira S., Shattock M.J., Sakata Y., Otsu K. (2019). Ablation of Toll-like receptor 9 attenuates myocardial ischemia/reperfusion injury in mice. Biochem. Biophys. Res. Commun..

[36] Scheen A.J. (2018). Does lower limb amputation concern all SGLT2 inhibitors?. Nat. Rev. Endocrinol..

[37] Behnammanesh G., Durante Z.E., Peyton K.J., Martinez-Lemus L.A., Brown S.M., Bender S.B., Durante W. (2019). Canagliflozin Inhibits Human Endothelial Cell Proliferation and Tube Formation. Front. Pharmacol..

[38] Krogmann A.O., Lüsebrink E., Steinmetz M., Asdonk T., Lahrmann C., Lütjohann D., Nickenig G., Zimmer S. (2016). Proinflammatory Stimulation of Toll-Like Receptor 9 with High Dose CpG ODN 1826 Impairs Endothelial Regeneration and Promotes Atherosclerosis in Mice. PLoS ONE.

[39] He B., Yang X., Li Y., Huang D., Xu X., Yang W., Dai Y., Zhang H., Chen Z., Cheng W.W. (2018). TLR9 (Toll-Like Receptor 9) Agonist Suppresses Angiogenesis by Differentially Regulating VEGFA (Vascular Endothelial Growth Factor A) and sFLT1 (Soluble Vascular Endothelial Growth Factor Receptor 1) in Preeclampsia. Hypertension.

[40] Zhao D., Liu H., Dong P. (2019). Empagliflozin reduces blood pressure and uric acid in patients with type 2 diabetes mellitus: A systematic review and meta-analysis. J. Hum. Hypertens..

[41] Abdurrachim D., Teo X.Q., Woo C.C., Chan W.X., Lalic J., Lam C.S.P., Lee P.T.H. (2019). Empagliflozin reduces myocardial ketone utilization while preserving glucose utilization in diabetic hypertensive heart disease: A hyperpolarized 13 C magnetic resonance spectroscopy study. Diabetes Obes. Metab..

[42] Rena G., Hardie D.G., Pearson E.R. (2017). The mechanisms of action of metformin. Diabetologia.

[43] Duca F.A., Côté C.D., Rasmussen B.A., Zadeh-Tahmasebi M., Rutter G.A., Filippi B.M., Lam T.K. (2015). Metformin activates a duodenal Ampk–dependent pathway to lower hepatic glucose production in rats. Nat. Med..

[44] Kaizuka T., Morishita H., Hama Y., Tsukamoto S., Matsui T., Toyota Y., Kodama A., Ishihara T., Mizushima T., Mizushima N. (2016). An Autophagic Flux Probe that Releases an Internal Control. Mol. Cell.

[45] Zhang J., Wang J., Ng S., Lin Q., Shen H.-M. (2014). Development of a novel method for quantification of autophagic protein degradation by AHA labeling. Autophagy.

[46] Wang C.-Y., Shie S.-S., Hsieh I.-C., Tsai M.-L., Wen M.-S. (2015). FTO modulates circadian rhythms and inhibits the CLOCK-BMAL1-induced transcription. Biochem. Biophys. Res. Commun..

