# Supplementary material for: TLR9 Binding to Beclin 1 and Mitochondrial SIRT3 by a Sodium-Glucose Co-Transporter 2 Inhibitor Protects the Heart from Doxorubicin Toxicity
Source: Biology (Basel). 2020 Oct 29;9(11):369. doi: 10.3390/biology9110369 (PMC7693736; doi:10.3390/biology9110369)
Supplement: Supplementary file 1 [file biology-09-00369-s001.pdf]

biology

MDPI

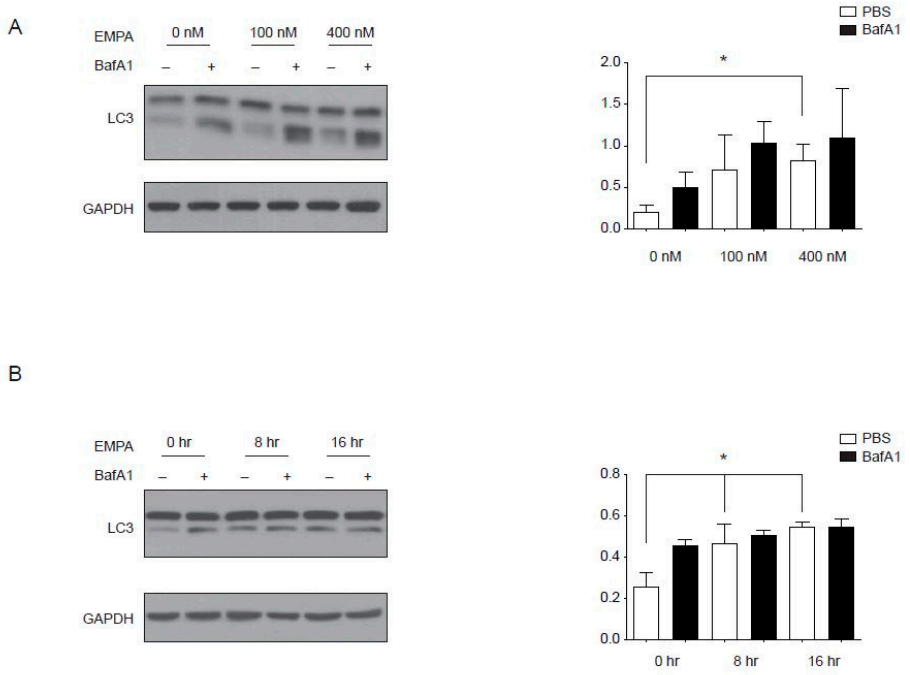

Figure S1. Sodium-glucose co-transporter 2 inhibition increases the autophagic flux in cardiomyocytes. A. Dose-dependent changes in LC3-II/I ratio after empagliflozin treatment in neonatal cardiomyocytes with or without BafA1 (n = 3 per group. * $p < 0.05$, data were analyzed by one-way analysis of variance (ANOVA) with Dunnett post hoc analysis). **B.** Time-dependent changes in LC3-II/I ratio after empagliflozin treatment in neonatal cardiomyocytes with or without BafA1 (n = 3 per group. * $p < 0.05$, data were analyzed by one-way analysis of variance (ANOVA) with Dunnett post hoc analysis).

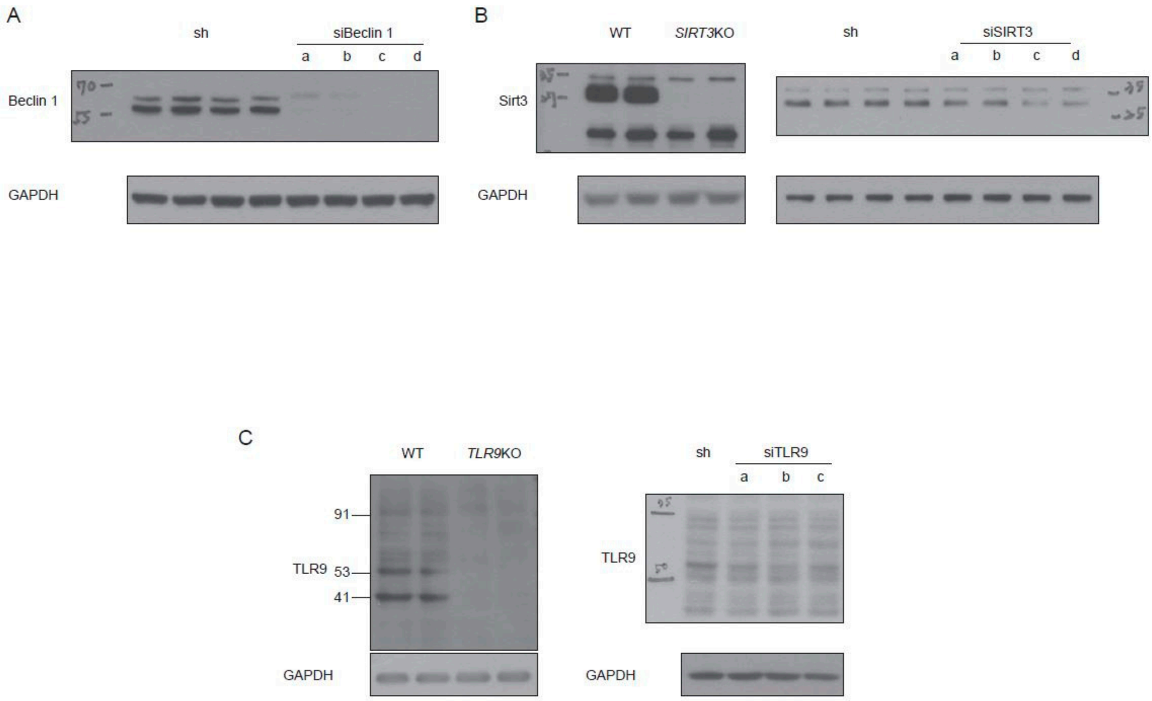

Figure S2. Verification of antibody and efficiency of siBeclin 1, siSirt3, and siTLR9. A. Beclin 1 blotting of cardiomyocytes with sh or siBeclin 1 knockdown treatments. **B.** Left: SIRT3 blotting of hearts from wild-type (WT) and *SIRT3*KO mice. Right: SIRT3 blotting of cardiomyocytes with sh or siSIRT3 knockdown treatments. **C.** Left: TLR9 blotting of hearts from wild-type (WT) and *TLR9*KO mice. Right: TLR9 blotting of cardiomyocytes with sh or siTLR9 knockdown treatments.

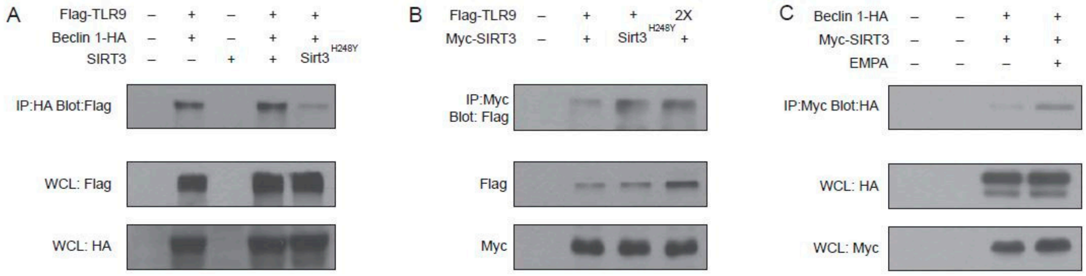

Figure S3. SIRT3 increased Beclin 1 and TLR9 bindings. A. Coimmunoprecipitation of Beclin 1 with TLR9 or TLR9 with Beclin 1 from 293 cells with or without empagliflozin (200 nM) treatment. Western blot analysis of 293 whole-cell extracts transfected with Flag-TLR9 and Beclin 1-HA and immunoprecipitated with Beclin 1-HA (lane 1) or Flag-TLR9 (lane 2). **B.** Coimmunoprecipitation of Beclin 1-HA with Flag-TLR9 from 293 cells with SIRT3 or SIRT3^{H248Y}. Western blot analysis of 293

whole-cell extracts transfected with Flag-TLR9 and Beclin 1-HA with SIRT3 or SIRT3^{H248Y} and immunoprecipitated with HA-Beclin 1 (lane 1). **C.** TLR9 interacted with SIRT3 *in vitro*. Flag-TLR9, Myc-SIRT3, and Myc-SIRT3^{H248Y} were immunoprecipitated and prepared from 293 cells and used for *in vitro* binding assay. The interactions were analyzed by immunoblotting for Flag-tagged proteins. **D.** Coimmunoprecipitation of Beclin 1-HA with Myc-SIRT3 in 293 cells with or without empagliflozin (200 nM) treatment. Western blot analysis of 293 whole-cell extracts transfected with HA-Beclin 1 and Myc-SIRT3 and immunoprecipitated with Myc-SIRT3 (lane 1).

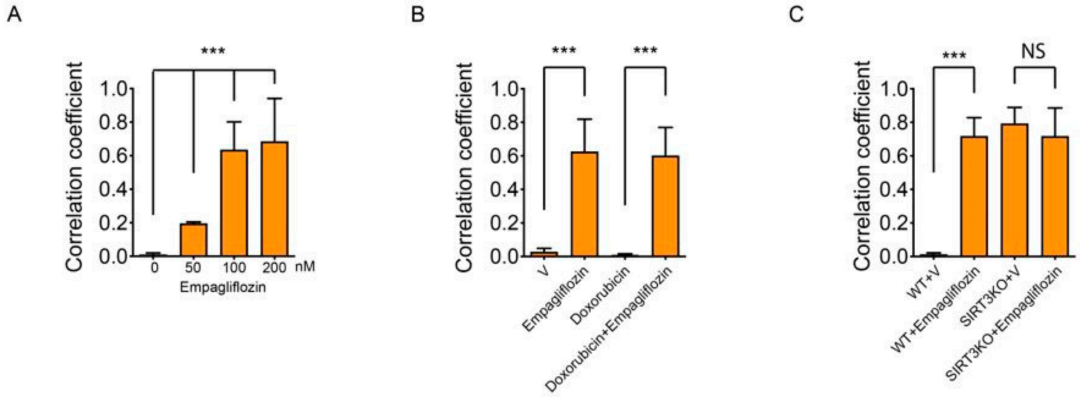

Figure S4. Colocalization analysis of overexpressed GFP-TLR9 and mitochondria stained with Mitotracker Deep Red FM. A. AC16 human cardiomyocytes stained for mitochondria and overexpressed GFP-TLR9. Cells were treated with indicated doses of empagliflozin before imaging and analyzed with Pearson's correlation coefficients of images of red mitochondria and GFP-TLR9 in AC16 human cardiomyocytes. ($n = 4$ experiments and 100 cells per experiment, *** $p < 0.001$, data were analyzed by the one-way ANOVA). **B.** AC16 human cardiomyocytes stained for mitochondria and overexpressed GFP-TLR9. Cells were treated with 200nM empagliflozin or 1 μM doxorubicin before imaging and analyzed with Pearson's correlation coefficients of images of red mitochondria and GFP-TLR9 in AC16 human cardiomyocytes. ($n = 4$ experiments and 100 cells per experiment, *** $p < 0.001$, data were analyzed by the two-way analysis of variance (ANOVA) with Tukey post hoc analysis). **C.** WT and SIRT3 KO cells stained for mitochondria (red) and GFP-TLR9. Cells were treated with 200 nM empagliflozin before imaging and analyzed with Pearson's correlation coefficients of images of red mitochondria and GFP-TLR9 in AC16 human cardiomyocytes. (n = 4 experiments and 100 cells per experiment, *** $p < 0.001$, NS, not significant, data were analyzed by the two-way analysis of variance (ANOVA) with Tukey post hoc analysis).

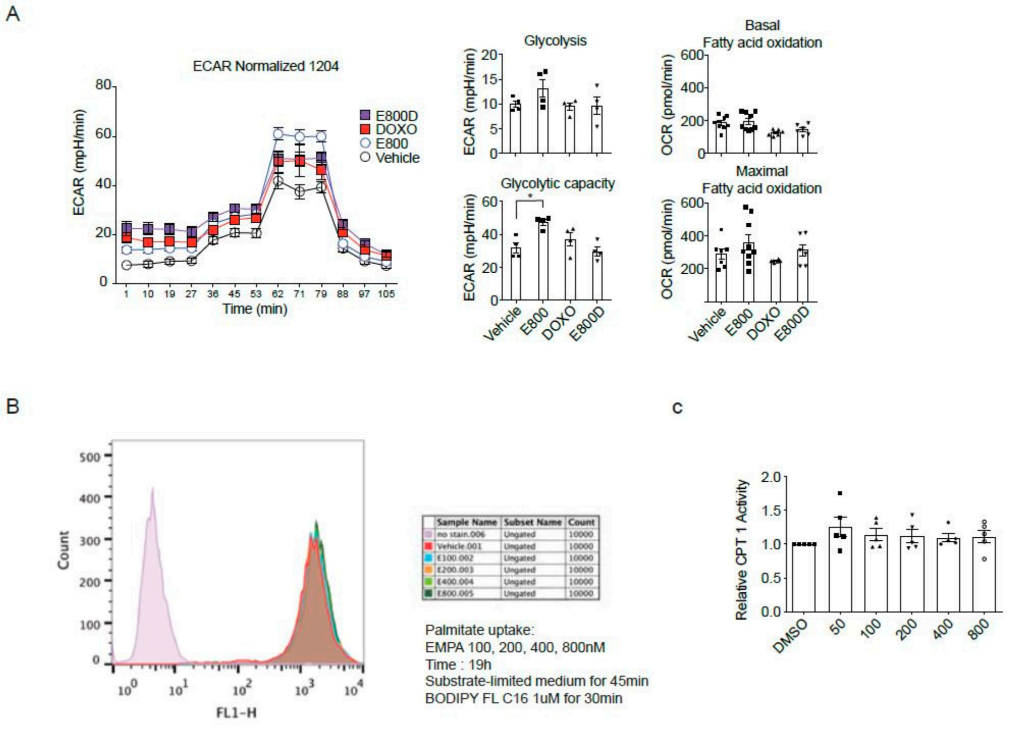

Figure S5. Glycolysis, Fatty acid uptake, and CPT 1 activity in cardiomyocytes after empagliflozin treatment. A. Glycolysis, basal fatty acid oxidation, glycolytic capacity, and maximal fatty acid oxidation analyzed by Seahorse instruments (n = 4~9, * $p < 0.05$, data were analyzed by the one-way ANOVA with Tukey post hoc analysis). **B.** Palmitate uptake in the neonatal cardiomyocytes after empagliflozin treatments analyzed by BODIPY FL C_{16} lipid probes. **C.** Carnitine acyltransferase activity in neonatal cardiomyocytes after empagliflozin treatment (n = 5, data were analyzed by the one-way ANOVA with Tukey post hoc analysis).

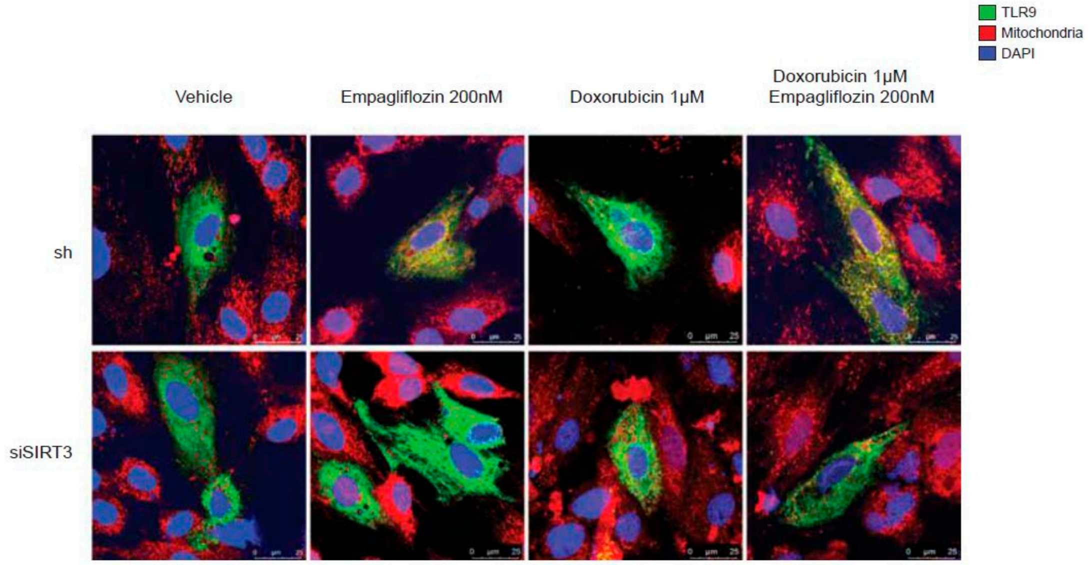

Figure S6. SIRT3 knockdown and TLR9 abundances after empagliflozin treatment in cardiomyocytes. Images of neonatal cardiomyocytes treated with sh or siSIRT3 RNA and stained for

mitochondria (red), nucleus (blue), and TLR9 (green). Cells were treated with empagliflozin (200 nM) or doxorubicin (1μM) before imaging.

Table S1. siRNA and Real time PCR primer sequences.

Gene	Primer Sequence
Rat *Beclin 1* siRNA	5'-CGUACAGGAUGGACGUGGA-3'
rat *TLR9* siRNA 1	5'-AACCUGAGCUAUAACGGUA-3'
rat *TLR9* siRNA 2	5'-CUGACUGGGUGUAUAACGA-3'
human *TLR9* siRNA 1	5'- CGGCAACUGUUAUUACAAG-3'
human *TLR9* siRNA 2	5'-ACAAUAAGCUGGACCUCUA-3'
human *SIRT3* siRNA 1	5'- UUGAGAGAGUGUCGGGCAU-3'
human *SIRT3* siRNA 2	5'-GGACCAGACAAAUAGGAUG-3'.
mBNP, Forward	5′-CAGCTCTTGAAGGACCAAGG-3′
mBNP, Reverse	5′-AGACCCAGGCAGAGTCAGAA-3′;
mCol1a1, Forward	5'-GCTCCTCTTAGGGGCCACT-3'
mCol1a1, Reverse	5'-ATTGGGGACCCTTAGGCCAT-3'
mTP53, Forward	5'-CTCTCCCCCGCAAAAGAAAAA-3'
mTP53, Reverse	5'-CGGAACATCTCGAAGCGTTA-3'

Publisher's Note: MDPI stays neutral with regard to jurisdictional claims in published maps and institutional affiliations.

© 2020 by the author. Licensee MDPI, Basel, Switzerland. This article is an open access article distributed under the terms and conditions of the Creative Commons Attribution (CC BY) license (http://creativecommons.org/licenses/by/4.0/).
